# Syntheses of dibenzo[*d*,*d*']benzo[2,1-*b*:3,4-*b*']difuran derivatives and their application to organic field-effect transistors

**DOI:** 10.3762/bjoc.12.79

**Published:** 2016-04-26

**Authors:** Minh Anh Truong, Koji Nakano

**Affiliations:** 1 Department of Organic and Polymer Materials Chemistry, Tokyo University of Agriculture and Technology, 2-24-16 Naka-cho, Koganei, Tokyo 184-8588, Japan

**Keywords:** furan, heteroacenes, organic field-effect transistors, organic semiconductor

## Abstract

Ladder-type π-conjugated compounds containing a benzo[2,1-*b*:3,4-*b*']difuran skeleton, such as dibenzo[*d*,*d*']benzo[2,1-*b*:3,4-*b*']difuran (*syn*-DBBDF) and dinaphtho[2,3-*d*:2',3'-*d*']benzo[2,1-*b*:3,4-*b*']difuran (*syn*-DNBDF) were synthesized. Their photophysical and electrochemical properties were revealed by UV–vis absorption and photoluminescence spectroscopy and cyclic voltammetry. Organic field-effect transistors (OFETs) were fabricated with these compounds as organic semiconductors, and their semiconducting properties were evaluated. OFETs with *syn*-DBBDF and *syn*-DNBDF showed typical p-type characteristics with hole mobilities of <1.5 × 10^−3^ cm^2^·V^−1^·s^−1^ and <1.0 × 10^−1^ cm^2^·V^−1^·s^−1^, respectively.

## Introduction

Organic semiconductors have significantly been developed in the past two decades by virtue of their advantages, such as low weight, flexibility, large-area processability, which are different features from conventional silicon-based semiconductors. Organic semiconducting materials can be used as active layers in organic field-effect transistors (OFETs) [1–7], organic light-emitting diodes (OLEDs) [8–10], and organic photovoltaics (OPVs) [11–12]. Among many organic semiconducting materials so far reported, thiophene-fused π-conjugated compounds have been widely studied as organic semiconducting materials and found to exhibit high semiconducting performances [5,13–16].

**Figure 1:**
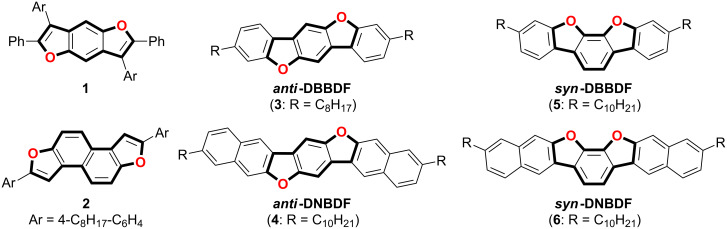
Structures of furan-fused ladder-type π-conjugated compounds.

Furan-containing π-conjugated compounds have attracted less attention until recently [17–27]. The oxygen atom possesses a smaller van der Waals radius than a sulfur atom. Accordingly, furan-containing π-conjugated compounds should be expected to form a denser packing structure in the solid state, which is one of the main requirements for high semiconducting properties [28–31]. In 2007, Nakamura and co-workers reported the synthesis of furan-fused ladder-type π-conjugated compounds, benzo[1,2-*b*:4,5-*b*']difurans (BDFs) **1** and their application to OLEDs as hole-transporting materials (Figure 1) [32]. They also synthesized a series of isomeric BDFs (benzo[1,2-*b*:5,4-*b*']difurans and benzo[1,2-*b*:6,5-*b*']difurans) and studied their structure–property relationship [33–34]. Furthermore, naphthodifurans with a fused-naphthalene between two furan rings have been developed as organic semiconductors for OFETs [19–20]. In particular, the naphtho[2,1-*b*:6,5-*b*']difuran derivative **2** has been reported to demonstrate an excellent OFET mobility of 3.6 cm^2^·V^−1^·s^−1^ [19]. Previously, we have reported the synthesis of dibenzo[*d*,*d*']benzo[1,2-*b*:4,5-*b*']difurans (*anti*-DBBDFs), which is also a π-extended homologue of BDF [35]. The OFET devices with an *anti*-DBBDF skeleton exhibited p-type semiconducting properties [36–37]. For example, dialkyl-substituted *anti*-DBBDF **3** showed a hole mobility of 0.042 cm^2^·V^−1^·s^−1^ [38]. Recently, we have also found that dinaphtho[2,3-*d*:2',3'-*d*']benzo[1,2-*b*:4,5-*b*']difuran (*anti*-DNBDF **4**) with a more extended π-conjugation afforded higher hole mobility of 0.33 cm^2^·V^−1^·s^−1^ [39–41]. These studies clearly demonstrate that furan-fused π-conjugated compounds are promising candidates as organic semiconducting materials, and it is highly desirable to investigate the structure−property relationship thoroughly for further development of furan-containing semiconducting materials.

Herein we report the synthesis of ladder-type π-conjugated compounds containing a benzo[2,1-*b*:3,4-*b*']difuran skeleton, such as dibenzo[*d*,*d*']benzo[2,1-*b*:3,4-*b*']difuran (*syn*-DBBDF **5**) and dinaphtho[2,3-*d*:2',3'-*d*']benzo[2,1-*b*:3,4-*b*']difuran (*syn*-DNBDF **6**, Figure 1) [42–46]. The physical and electrochemical properties of the synthesized compounds are also discussed. OFETs with these compounds as semiconducting layers were found to exhibit relatively high hole mobility of <1.0 × 10^−1^ cm^2^·V^−1^·s^−1^.

## Results and Discussion

### Synthesis

**Scheme 1:**
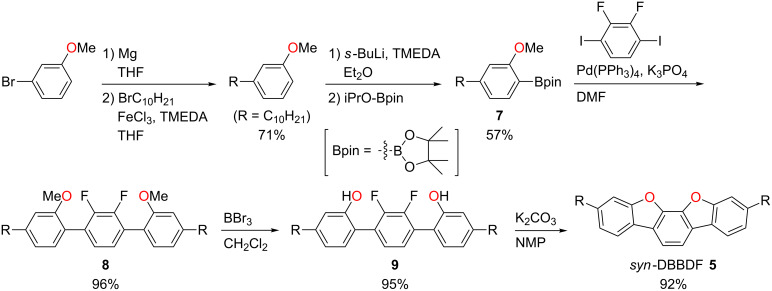
Synthesis of *syn*-DBBDF **5**.

**Scheme 2:**
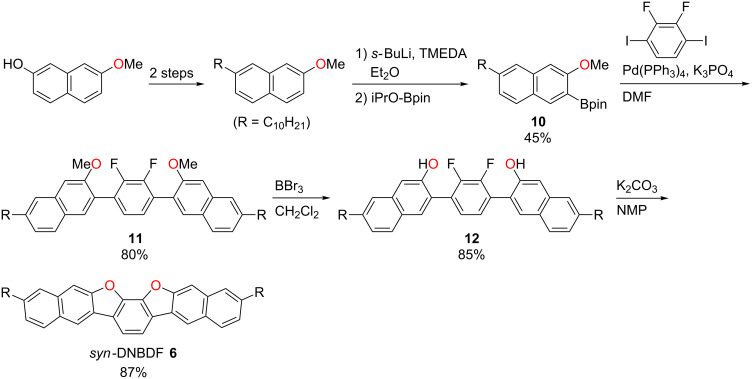
Synthesis of *syn*-DNBDF **6**.

The synthetic routes to *syn*-DBBDF **5** and *syn*-DNBDF **6** are described in Scheme 1 and Scheme 2. 3-Decylanisole was first synthesized from commercially available 3-bromoanisole via iron-catalyzed cross-coupling reaction with decylmagnesium bromide in 71% yield [47]. Lithiation of the obtained 3-decylanisole with *s-*BuLi and the following treatment with 2-isopropoxy-4,4,5,5-tetramethyl-1,3,2-dioxaborolane (iPrO-Bpin) gave boronate ester **7** in 57% yield. Then, terphenyl **9** was synthesized via palladium-catalyzed Suzuki–Miyaura cross coupling of boronate ester **7** with 2,3-difluoro-1,4-diiodobenzene (96% yield) and subsequent demethylation (95% yield). Finally, the desired *syn*-DBBDF **5** was successfully synthesized via the double intramolecular cyclization under basic conditions at high temperature (92% yield) [37,43]. The same synthetic strategy was applied to the synthesis of *syn*-DNBDF (Scheme 2). 2-Decyl-7-methoxynaphthalene was prepared from 7-methoxynaphthalen-2-ol in two steps according to the literature [23,48], and used for the synthesis of boronate ester **10** (45% yield). The following cross coupling (80% yield), demethylation (85% yield), and the double cyclization (87% yield) gave the target *syn*-DNBDF **6**. The obtained *syn*-DBBDF **5** is soluble in common organic solvents and can be purified by column chromatography. In contrast, because of low solubility in common organic solvents, the crude product of *syn*-DNBDF **6** was purified by washing several times with water and the subsequent sublimation.

### Thermal properties

**Figure 2:**
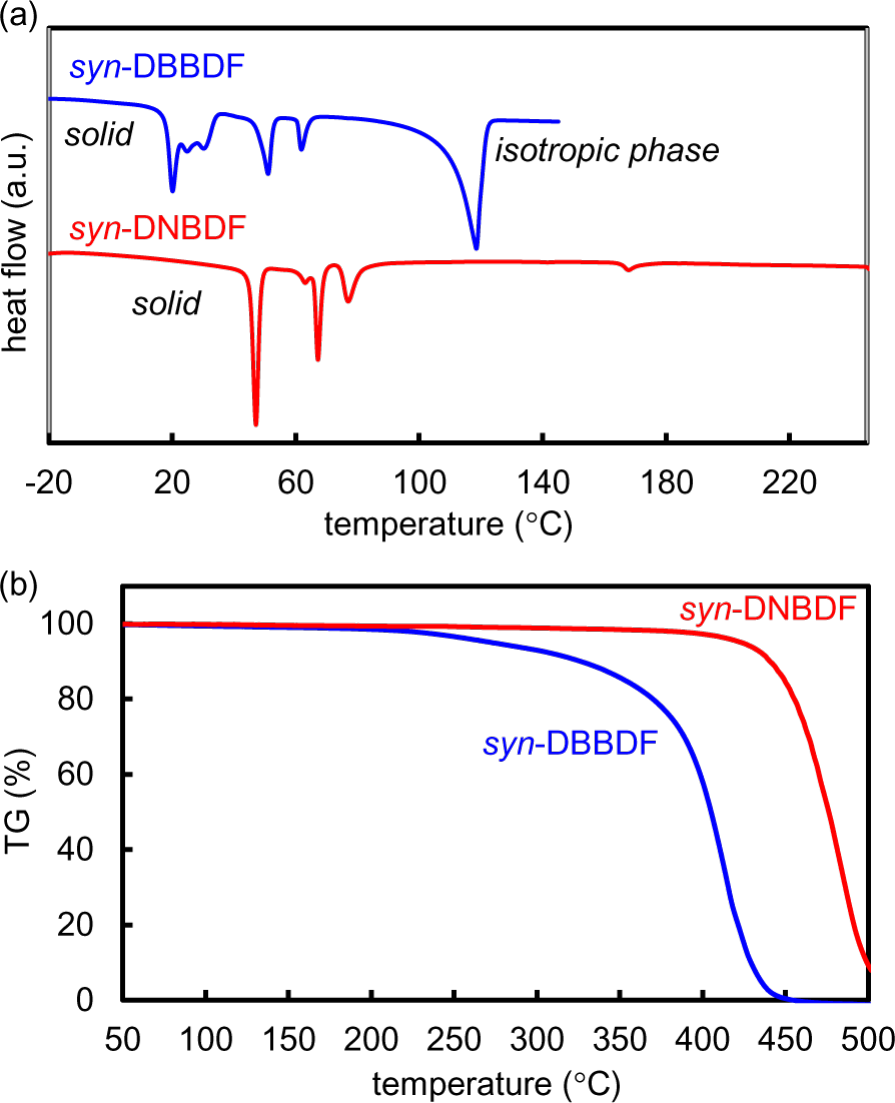
(a) DSC and (b) TG curves of *syn*-DBBDF **5** and *syn*-DNBDF **6**.

The phase-transition properties and thermal stability of *syn*-DBBDF **5** and *syn*-DNBDF **6** were evaluated by differential scanning calorimetry (DSC) and thermogravimetric analysis (TGA), respectively. The DSC scans of *syn*-DBBDF **5** and *syn*-DNBDF **6** showed some transition peaks with the first phase-transition temperature at 20 °C and 45 °C, respectively, in the heating process (Figure 2a). Such phase-transition temperatures are >50 °C lower than those of their *anti*-isomers **3** and **4** [38–39]. These results indicate that *syn*-DBBDF **5** and *syn*-DNBDF **6** form weaker intermolecular interactions in the solid state than their corresponding *anti*-isomers. The mesophase of *syn*-DBBDF **5** was converted to the isotropic phase at 115 °C, while *syn*-DNBDF **6** did not melt below 250 °C. From the TG measurement, the temperatures of 5% weight loss (*T*_d5_) of *syn*-DBBDF **5** and *syn*-DNBDF **6** were estimated to be 272 °C and 423 °C, respectively (Figure 2b).

### Photophysical properties

**Figure 3:**
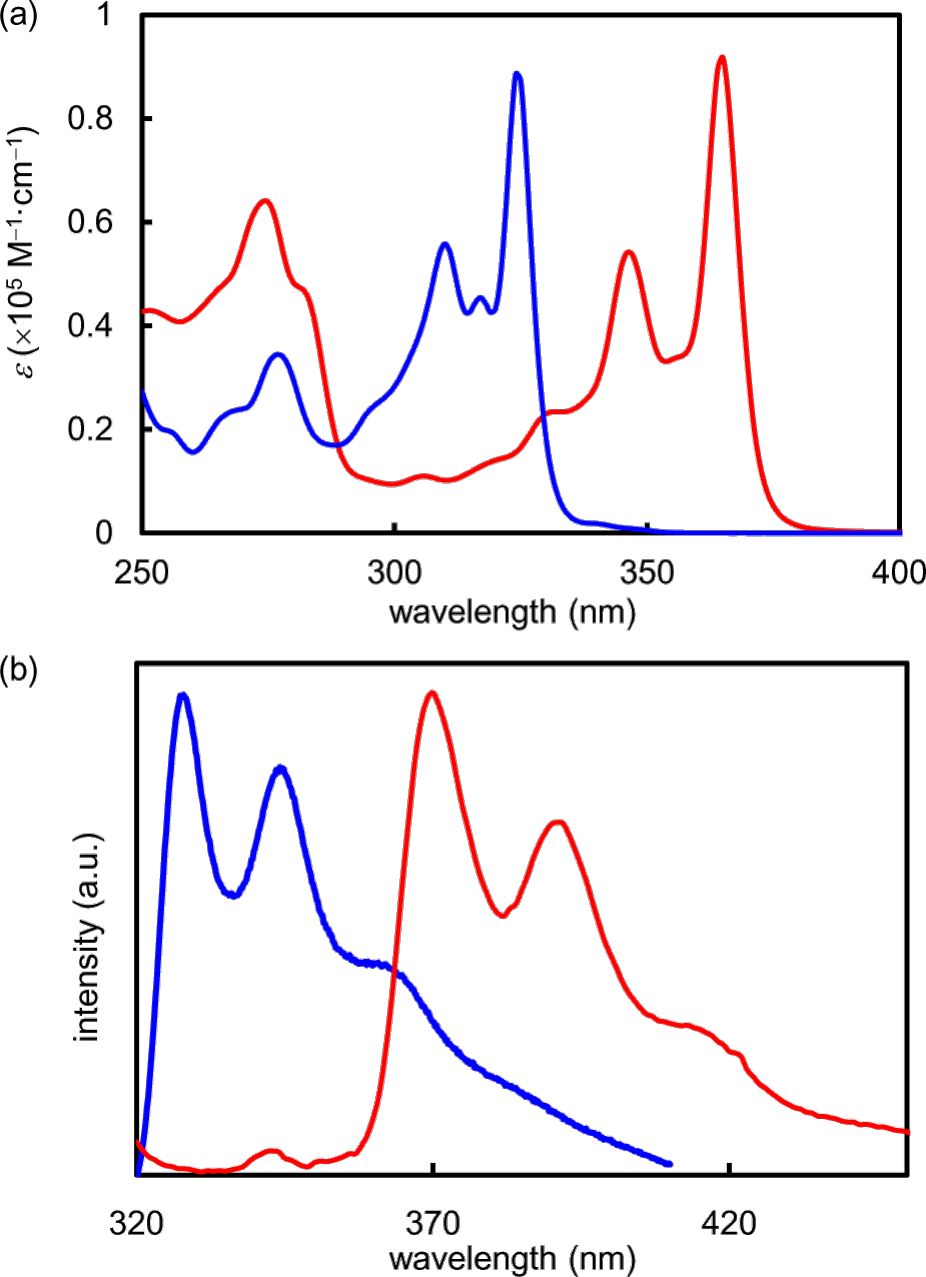
(a) UV–vis absorption spectra of *syn*-DBBDF **5** (blue line) and *syn*-DNBDF **6** (red line) in CHCl_3_ (1.0 × 10^−5^ M) and (b) normalized photoluminescence spectra of *syn*-DBBDF **5** (blue line) and *syn*-DNBDF **6** (red line) in CHCl_3_ (1.0 × 10^−7^ M).

The UV–vis spectrum of *syn*-DBBDF **5** in chloroform showed the strongest absorption maximum at 324 nm, while *syn*-DNBDF **6** showed a red-shifted absorption spectrum with the strongest absorption maximum at 365 nm (Figure 3a and Table 1). Since s*yn*-DNBDF **6** contains one more benzene ring at each terminal of the π-conjugated skeleton than *syn*-DBBDF **5**, it should possess an extended π-conjugation length, resulting in a red-shifted absorption spectrum. The HOMO–LUMO energy gaps estimated from the absorption edges were 3.72 eV and 3.32 eV for *syn*-DBBDF **5** and *syn*-DNBDF **6**, respectively. Their photoluminescence spectra as shown in Figure 3b exhibited mirror images of their absorption spectra with small Stokes shifts (376 cm^–1^ for *syn*-DBBDF **5**; 370 cm^–1^ for *syn*-DNBDF **6**), which reflect their high rigidity. Similar to its absorption spectra, *syn*-DNBDF **6** showed a red-shifted emission band with a relatively high quantum yield (Φ = 61% in CHCl_3_ solution).

**Table 1:** Photophysical and electrochemical properties of *syn*/*anti*-DBBDFs and DNBDFs.

Compound	λ_abs_ (nm)^a^	λ_em_ (nm)^b^	Φ (%)^c^	Stokes shift (cm^–1^)	*E*_g_ (eV)^d^	*E*_ox_^onset^ (V)^e^	*E*_HOMO_ (eV)^f^

*syn*-DBBDF **5**	324	328	18	376	3.72	0.84	−5.64
*syn*-DNBDF **6**	365	370	61	370	3.32	0.56	−5.36
*anti*-DBBDF **3**	342	–	–	–	3.51	–	–
*anti*-DNBDF **4**	394	–	–	–	3.15	–	–

^a^In CHCl_3_ (1.0 × 10^−5^ M). ^b^In CHCl_3_ (1.0 × 10^−7^ M). Excitation at 310 nm. ^c^Absolute quantum yield determined by a calibrated integrating sphere system. Excitation at 275 nm for *syn*-DBBDF **5** and *syn*-DNBDF **6**. ^d^Optical band gaps estimated from the onset position of the UV–vis absorption spectra in solution. ^e^Onset potentials (vs Fc/Fc^+^) of the first oxidation wave determined by cyclic voltammetry: 1.0 mM solution in CH_2_Cl_2_ (*syn*-DBBDF **5**) or Cl_2_CHCHCl_2_ (*syn*-DNBDF **6**) with 0.1 M Bu_4_NClO_4_, Pt as working and counter electrodes, scan rate = 50 mV·s^−1^. ^f^Calculated according to *E*_HOMO_ = −(*E*_ox_ + 4.80) eV (Fc/Fc^+^ redox couple: 4.8 eV below the vacuum level).

To investigate the structure–property relationship of DBBDFs and DNBDFs, the optical properties of *syn*-DBBDF **5** and *syn*-DNBDF **6** were compared with those of *anti*-DBBDF **3** and *anti*-DNBDF **4**. The UV–vis spectra of *anti*-DBBDF **3** and *anti*-DNBDF **4** were reported to show absorption maxima (342 nm for *anti*-DBBDF **3**; 394 nm for *anti*-DNBDF **4**) and absorption edges [353 nm (3.51 eV) for *anti*-DBBDF **3**; 410 nm (3.15 eV) for *anti*-DNBDF **4**] at longer wavelengths than *syn*-DBBDF **5** and *syn*-DNBDF **6**, respectively [38–39]. Accordingly, *syn*-isomers are indicated to possess shorter π-conjugation lengths than *anti*-isomers.

### Electrochemical properties

**Figure 4:**
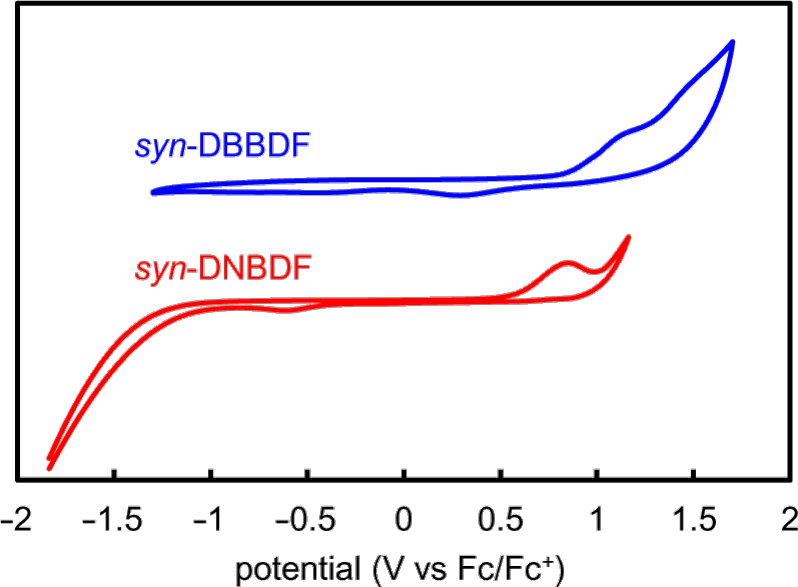
Cyclic voltammograms of *syn*-DBBDF **5** and *syn*-DNBDF **6** (measurement conditions: 1.0 mM in CH_2_Cl_2_ for *syn*-DBBDF **5** or Cl_2_CHCHCl_2_ for *syn*-DNBDF **6** with 0.1 M Bu_4_NClO_4_; Pt as working and counter electrodes; scan rate = 50 mV·s^−1^).

Cyclic voltammograms of *syn*-DBBDF **5** and *syn*-DNBDF **6** are shown in Figure 4 [1.0 mM solution in CH_2_Cl_2_ (*syn*-DBBDF **5**) or Cl_2_CHCHCl_2_ (*syn*-DNBDF **6**) with 0.10 M Bu_4_NClO_4_], and the electrochemical properties were summarized in Table 1. *syn*-DBBDF **5** exhibited two oxidation waves, and an onset potential of the first oxidation wave was determined to be 0.84 V (vs Fc/Fc^+^). Accordingly, the HOMO energy level was estimated to be −5.64 eV under the premise that the energy level of Fc/Fc^+^ is 4.8 eV below the vacuum level [49–51]. In contrast, *syn*-DNBDF **6** showed one oxidation wave with an onset potential of 0.56 eV (vs Fc/Fc^+^, HOMO = −5.36 eV). The lower oxidation potential and higher HOMO energy level of *syn*-DNBDF **6** should reflect its longer π-conjugation length than *syn*-DBBDF **5**. Based on their HOMO energy levels and HOMO−LUMO energy gaps, *syn*-DBBDF **5** and *syn*-DNBDF **6** are expected to work as stable semiconducting materials under ambient conditions.

### Fabrication of OFETs with *syn*-DBBDF- and *syn*-DNBDF-based thin films and evaluation of semiconducting properties

To study the semiconducting properties of *syn*-DBBDF **5** and *syn*-DNBDF **6**, bottom-gate/top-contact OTFTs were utilized as a device structure. Thin films of *syn*-DBBDF **5** and *syn*-DNBDF **6** were deposited by sublimation under high vacuum (*p* < 10^−5^ Pa) at a rate of ca. 1 Å·s^−1^ for *syn*-DBBDF and ca. 0.4 Å·s^−1^ for *syn*-DNBDF onto the Si/SiO_2_ substrates. The substrate temperature (*T*_sub_) during deposition has been known to have a great impact on the OTFT performance by affecting the nucleation and growth of the organic molecules [52–53]. Accordingly, the thin films were fabricated at different substrate temperatures. In addition to the bare Si/SiO_2_ substrates, the HMDS (hexamethyldisilazane)-treated substrates were used to evaluate the effect of the substrate structure on the device performance. The gold source/drain electrodes were deposited on the thin films. The channel width and length were 500 μm and 50 μm, respectively.

**Figure 5:**
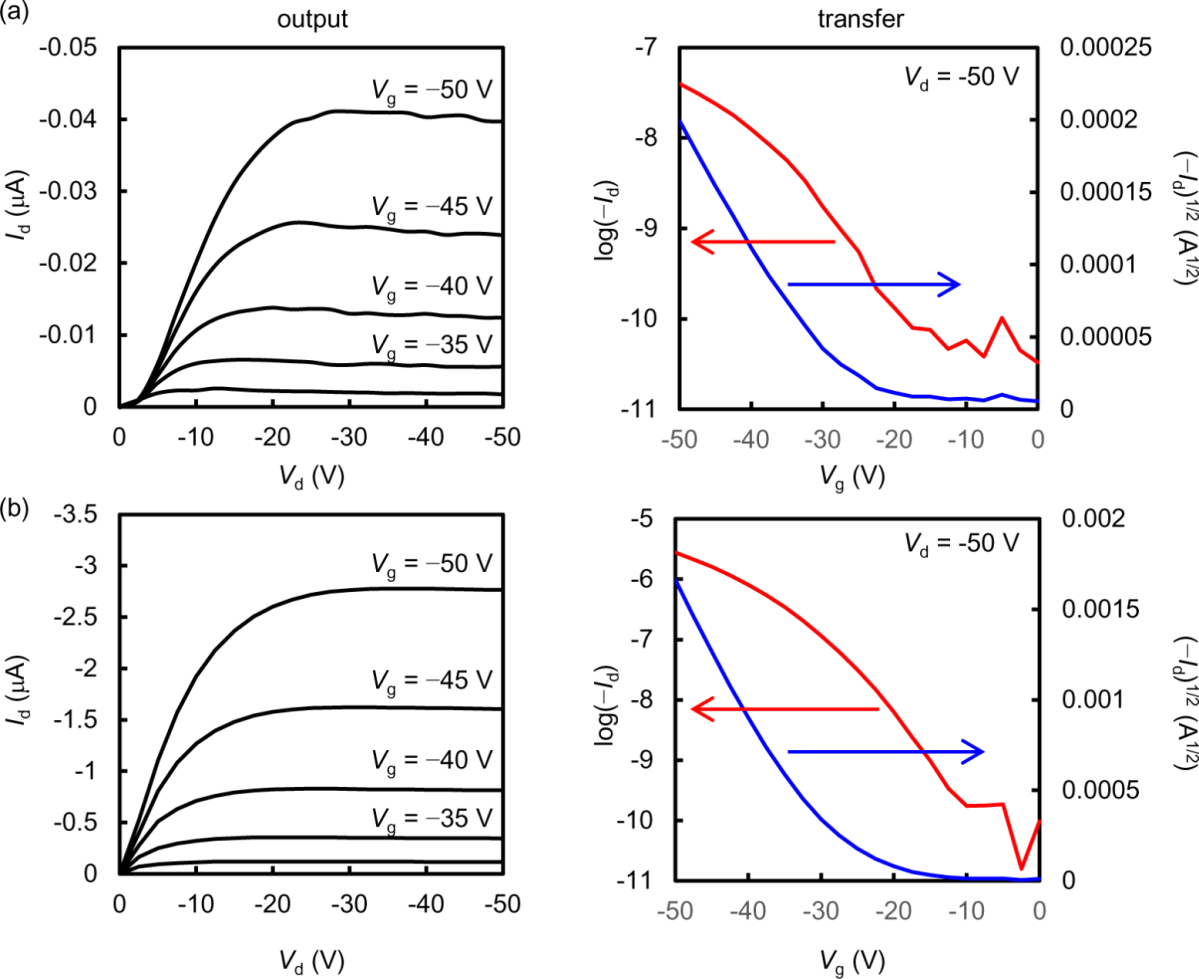
Output and transfer characteristics of the representative OFETs with a thin film of (a) *syn*-DBBDF **5** (*T*_sub_ = 30 °C) and (b) *syn*-DNBDF **6** (*T*_sub_ = 90 °C) on HMDS-treated Si/SiO_2_ substrates.

Both *syn*-DBBDF- and *syn*-DNBDF-based OFETs demonstrated typical p-type semiconducting characteristics. The extracted FET parameters and the transfer/output characteristics are summarized in Table 2, Figure 5, and Figure S21 (Supporting Information File 1). The *syn*-DBBDF-based OFETs fabricated on bare Si/SiO_2_ substrates at *T*_sub_ = 30 °C showed a field-effect mobility μ_FET_ of 5.0 × 10^−5^ cm^2^·V^−1^·s^−1^ and an *I*_on_/*I*_off_ ratio of 10^1^, while those with HMDS-treated substrates demonstrated higher mobility of 1.5 × 10^−3^ cm^2^·V^−1^·s^−1^ with an *I*_on_/*I*_off_ ratio of 10^3^. The deposition of *syn*-DBBDF **5** at *T*_sub_ = 60 °C did not give a thin film, which should be caused by re-sublimation of *syn*-DBBDF **5** from the surface. The more π-extended *syn*-DNBDF **6** afforded higher performances than *syn*-DBBDF **5**. OFETs fabricated on the bare and HMDS-treated Si/SiO_2_ substrates at *T*_sub_ = 30 °C showed a field-effect mobility of 2.3 × 10^−2^ cm^2^·V^−1^·s^−1^ (*I*_on_/*I*_off_ = 10^3^) and 2.0 × 10^−2^ cm^2^·V^−1^·s^−1^ (*I*_on_/*I*_off_ = 10^3^), respectively. The FET performance also depends on the substrate temperature during thin-film fabrication. Thus, the highest hole mobility of 1.0 × 10^−1^ cm^2^·V^−1^·s^−1^ was obtained for the *syn*-DNBDF-based device fabricated on the HMDS-treated substrate at *T*_sub_ = 90 °C, while it was lower than that fabricated with *anti*-DNBDF derivatives [39].

**Table 2:** FET characteristics.

Compound	Surfactant	*T*_sub_ (°C)	μ_FET_ (cm^2^·V^−1^·s^−1^)	*V*_th_ (V)	*I*_on_/*I*_off_

*syn*-DBBDF **5**	–	30	5.0 × 10^−5^	−26	10^1^
	HMDS	30	1.5 × 10^−3^	−25	10^3^
*syn*-DNBDF **6**	–	30	2.3 × 10^−2^	−24	10^3^
	–	90	6.5 × 10^−2^	−25	10^4^
	HMDS	30	2.0 × 10^−2^	−22	10^3^
	HMDS	90	1.0 × 10^−1^	−28	10^5^

### Analysis of thin films

**Figure 6:**
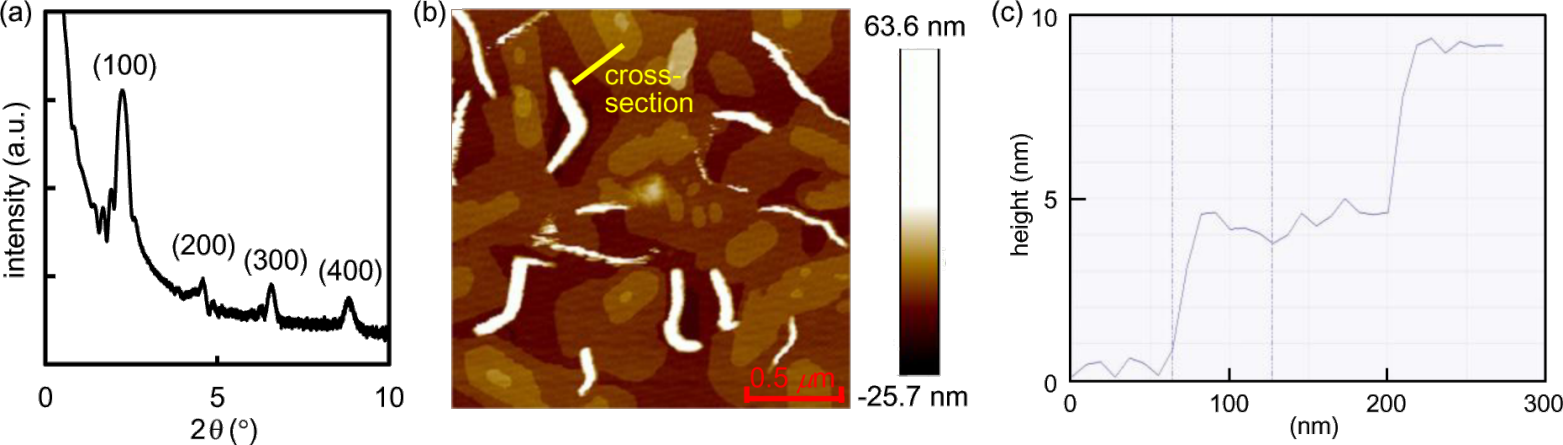
(a) XRD pattern, (b) AFM image (2 × 2 μm), and (c) cross-section height of a thin film of *syn*-DNBDF **6** on HMDS-treated Si/SiO_2_ substrates (*T*_sub_ = 90 °C).

The vapor-deposited thin films of *syn*-DBBDF **5** and *syn*-DNBDF **6** were analyzed by X-ray diffraction (XRD) and atomic force microscopy (AFM). Figure 6 shows the out-of-plane XRD pattern and an AFM image of the thin film of *syn*-DNBDF **6** on the HMDS-treated Si/SiO_2_ substrate (*T*_sub_ = 90 °C), which demonstrated the highest mobility in this study. The layer structure was confirmed with a monolayer thickness (*d*-spacing) of 3.94 nm (2θ = 2.24°). Molecular lengths with extended linear alkyl chains are expected to be ca. 4.2 nm. Accordingly, *syn*-DNBDF **6** should be arranged on the substrate with its molecular long axis almost perpendicular to the substrate. Such a layer structure was also confirmed by AFM. As shown in Figure 6b,c, the thin film of *syn*-DNBDF **6** forms relatively large grains (ca. 0.5 μm in size) with a layer structure (step heights ca. 4.0 nm) along with heterogeneous protrusions. The molecular arrangement indicated by these observations is advantageous for the in-plane charge transfer of OFETs. Based on XRD patterns and AFM images, the substrate treatment and the substrate temperature seem to have a limited impact on the molecular arrangement (Figures S22 and S23, Supporting Information File 1). The similar layer structure was also confirmed for *syn*-DBBDF **5** (Figures S22 and S23, Supporting Information File 1).

## Conclusion

In summary, we investigated the synthesis and properties of ladder-type π-conjugated compounds, dibenzo[*d*,*d*']benzo[2,1-*b*:3,4-*b*']difuran (*syn*-DBBDF **5**) and dinaphtho[2,3-*d*:2',3'-*d*']benzo[2,1-*b*:3,4-*b*']difuran (*syn*-DNBDF **6**). Based on the photophysical and electrochemical data, both compounds are expected to possess good air stability as organic semiconducting materials. The comparison with their *anti*-isomers revealed that the π-conjugation in *syn*-DBBDF **5** and *syn*-DNBDF **6** is less effective than those of their *anti*-isomers. OFETs based on these compounds were fabricated as bottom-gate top-contact devices, and their semiconducting properties were evaluated. All devices showed typical p-type transistor characteristics. The highest hole mobility of 1.0 × 10^−1^ cm^2^·V^−1^·s^−1^ was achieved when using *syn*-DNBDF-based OFET device.

## Supporting Information

File 1 General experimental procedures, synthetic procedures/characterization data of compounds **5**–**12**, device fabrication/evaluation procedures, OFET characteristics, XRD patterns, and AFM images.
