# Supplementary material for: Syntheses of dibenzo[d,d']benzo[2,1-b:3,4-b']difuran derivatives and their application to organic field-effect transistors
Source: Beilstein J Org Chem. 2016 Apr 26;12:805–12. doi: 10.3762/bjoc.12.79 (PMC4902046; doi:10.3762/bjoc.12.79)
Supplement: File 1 — General experimental procedures, synthetic procedures/characterization data of compounds 5–12, device fabrication/evaluation procedures, OFET characteristics, XRD patterns, and AFM images. [file Beilstein_J_Org_Chem-12-805-s001.pdf]

Supporting Information

for

Syntheses of dibenzo[*d*,*d*']benzo[2,1-*b*:3,4-*b*']difuran derivatives and their application to organic field-effect transistors

Minh Anh Truong and Koji Nakano*

Address: Department of Organic and Polymer Materials Chemistry, Tokyo University of Agriculture and Technology, 2-24-16 Naka-cho, Koganei, Tokyo 184-8588, Japan

Email: Koji Nakano - k_nakano@cc.tuat.ac.jp

* Corresponding author

General experimental procedures, synthetic procedures/characterization data of compounds 5–12, device fabrication/evaluation procedures, OFET characteristics, XRD patterns, and AFM images

Table of Contents

General procedures S2−S3

Synthetic procedures and characterization data of compounds **5–12** S4−S8

Device fabrication and evaluation procedures S9

^{1}H, ^{13}C, and ^{19}F NMR spectra of compounds **5–12** S10−S19

Output and transfer characteristics of OFET devices S20

XRD patterns and AFM images S21

References S22

General procedures. All manipulations involving air and/or moisture-sensitive compounds were carried out with standard Schlenk technique under argon. Reagents were used without further purification unless otherwise specified. Analytical thin-layer chromatography was performed on glass plates coated with 0.25 mm 230–400 mesh silica gel containing a fluorescent indicator. Column chromatography was performed by using silica gel (spherical neutral, particle size 63–210 μm).

NMR spectra were recorded in $CDCl_3$ or 1,1,2,2-tetrachloroethane-d_2 on a 500 MHz spectrometer (^{13}C 126 MHz), a 400 MHz spectrometer (^{1}H 400 MHz; ^{13}C 101 MHz, ^{19}F 376 MHz), or a 300 MHz spectrometer (^{1}H 300 MHz). Chemical shifts are reported in ppm relative to the internal standard signal (0 ppm for Me_4Si in $CDCl_3$) for ^{1}H and the residual solvent signal (77.16 ppm for $CDCl_3$; 73.8 ppm for 1,1,2,2-tetrachloroethane-d_2 [1]) for ^{13}C. Data are presented as follows: chemical shift, multiplicity (s = singlet, d = doublet, t = triplet, m = multiplet and/or multiple resonances), coupling constant in hertz (Hz), and signal area integration in natural numbers. Melting points were determined on a melting point apparatus. High resolution mass spectra are taken by atmospheric pressure chemical ionization time-of-flight (APCI−TOF) or matrix-assisted laser desorption/ionization time-of-flight (MALDI−TOF) method. Elemental analyses were performed at the A Rabbit Science Co., Ltd. Phase-transition behavior was analyzed by DSC apparatus. Heating rates were 10 °C per minute. Thermal decomposition was analyzed by TG−DTA apparatus. Heating rates were 10 °C per minute. Thermal decomposition temperatures T_{d5} were defined as temperature of 5% weight loss. UV-Vis absorption spectra were recorded on a UV−Vis scanning spectrophotometer. Photoluminescence spectra were recorded on a spectrofluorometer. Absolute quantum yields were determined by calibrated integrating sphere system. Cyclic voltammetric measurements were performed with an electrochemical analyzer in CH_2Cl_2 or $Cl_2CHCHCl_2$ containing 1.0 mM of substrate and 0.1 M of Bu_4NClO_4 as supporting electrolyte at a scan rate of 50 mV·s^{-1}. The counter and working electrodes were Pt wires, and the reference electrode was Ag/AgCl. All the potentials were calibrated with the standard ferrocene/ferrocenium redox couple (evaluated as the average of E_{pa} and E_{pc}) [2-4]. X-ray diffraction

was recorded on an X-ray diffractometer at room temperature using Cu Kα radiation (λ = 1.5406 Å) with a θ-2θ configuration. Atomic force microscope (AFM) experiments were performed in the tapping mode under ambient conditions.

Synthetic procedures and characterization data

2-(4-Decyl-2-methoxyphenyl)-4,4,5,5-tetramethyl-1,3,2-dioxaborolane (7): A 250 mL round-bottomed flask was charged with 3-decylanisole (1.09 g, 4.4 mmol), *N*,*N*,*N′*,*N′*-tetramethylethylenediamine (2.0 mL, 13.4 mmol), and Et_2O (66 mL) under argon. The mixture was cooled to −78 °C with a dry ice/acetone bath. *s*-BuLi (7.5 mL of 1.06 M hexane solution, 8.0 mmol) was added dropwise at −78 °C. After the reaction mixture was stirred at the same temperature over 2 h, 2-isopropoxy-4,4,5,5-tetramethyl-1,3,2-dioxaborolane (2.9 mL, 14.3 mmol) was added dropwise. The resulting mixture was gradually warmed to room temperature and stirred for 16 h. Water was added to the reaction mixture, and the organic layer was separated. The aqueous layer was extracted with Et_2O, and the combined organic layers were dried over Na_2SO_4, filtered, and concentrated under reduced pressure. The resulting crude residue was purified by silica gel column chromatography by using hexane/AcOEt (5:1) and 1% Et_3N as an eluent to afford 0.92 g (57% yield) of the title compound as a colorless oil: ^{1}H NMR (400 MHz, $CDCl_3$) δ = 7.59 (d, *J* = 7.3 Hz,1H), 6.77 (d, *J* = 7.3 Hz, 1H), 6.67 (s, 1H), 3.83 (s, 3H), 2.59 (t, *J* = 8 Hz, 2H), 1.62−1.57 (m, 2H), 1.34−1.25 (m, 26H), 0.88 (t, *J* = 6.9 Hz, 3H); ^{13}C NMR (101 MHz, $CDCl_3$) δ = 164.5, 148.2, 136.9, 120.5, 114.8, 110.9, 83.3, 55.9, 36.5, 32.0, 31.4, 29.7, 29.6, 29.4, 24.9, 22.8, 14.2; HRMS−APCI$^+$ (*m*/*z*) calcd for $C_{23}H_{40}BO_3$ ($[M + H]^+$) 375.3065, found 375.3070.

2',3'-Difluoro-2,2''-dimethoxy-4,4''-didecyl-1,1':4',1''-terphenyl (8): A 50 mL Schlenk tube was charged with **7** (0.96 g, 2.6 mmol), 2,3-difluoro-1,4-diiodobenzene (0.45 g, 1.2 mmol), $Pd(PPh_3)_4$ (71 mg, 61 μmol), K_3PO_4 (1.3 g, 6.1 mmol), and DMF (10 mL) under argon. The reaction mixture was degassed by three freeze−thaw pump cycles, and the tube was filled with argon. The reaction mixture was stirred at 100 °C for 66 h, diluted with $CHCl_3$, and washed with water. The organic layer was dried over Na_2SO_4, filtered, and concentrated under reduced pressure. The resulting crude residue was purified by silica gel column chromatography by using hexane/EtOAc (5:1) (R_f = 0.7) as an eluent to

afford 0.72 g (96% yield) of the title compound as a colorless solid: mp 81−85 °C; ^{1}H NMR (400 MHz, $CDCl_3$) δ = 7.21 (d, J = 7.8 Hz, 2H), 7.12(s, 2H), 6.87 (d, J = 7.8 Hz, 2H), 6.83 (s, 2H), 3.84 (s, 6H), 2.66 (t, J = 7.6 Hz, 4H), 1.71−1.64 (m, 4H), 1.36−1.28 (m, 28H), 0.89 (t, J = 6.2 Hz, 6H); ^{13}C NMR (101 MHz, $CDCl_3$) δ = 156.9, 148.5 (dd, J = 250.2, 15.3 Hz), 145.2 131.2, 127.1 (dd, J = 9.1, 4.8 Hz), 125.6, 121.5, 120.7, 111.5, 55.8, 36.4, 32.1, 31.6, 29.79, 29.77, 29.70, 29.6, 29.5, 22.9, 14.3; ^{19}F NMR (376 MHz, $CDCl_3$) δ = −139.1; HRMS−APCI$^+$ (m/z) calcd for $C_{40}H_{57}F_2O_2$ ([M + H]$^+$) 607.4321, found 607.4327.

4,4''-Didecyl-2',3'-difluoro-[1,1':4',1''-terphenyl]-2,2''-diol (9): A 50 mL Schlenk tube was charged with compound **8** (0.64 g, 1.1 mmol) and CH_2Cl_2 (10 mL) under argon. To the solution was added BBr_3 (7.0 mL of 1 M CH_2Cl_2 solution) at 0 °C dropwise, and the reaction mixture was stirred at 25 °C for 18 h. After the addition of iced water and saturated aqueous Na_2CO_3, the resulting mixture was extracted with CH_2Cl_2. The combined organic layers were washed with brine, dried over Na_2SO_4, filtered, and concentrated under reduced pressure. The resulting crude residue was purified by silica gel column chromatography by using hexane/EtOAc (3:1) (R_f = 0.6) as an eluent to afford 0.57 g (95% yield) of the title compound as a colorless solid: mp 134−137 °C; ^{1}H NMR (400 MHz, $CDCl_3$) δ = 7.18−7.15 (m, 4H), 6.84 (d, J = 7.8 Hz, 2H), 6.78 (s, 2H), 4.97 (s, 2H), 2.58 (t, J = 7.8 Hz, 4H), 1.66−1.58 (m, 4H), 1.31−1.25 (m, 28H), 0.86 (t, J = 6.6 Hz, 6H); ^{13}C NMR (101 MHz, $CDCl_3$) δ = 152.7, 148.8 (dd, J = 252.1, 15.3 Hz), 145.9, 131.0, 126.6 (dd, J = 7.7, 4.8 Hz), 126.3, 121.4, 118.6, 116.3, 35.9, 32.1, 31.3, 29.79, 29.75, 29.7, 29.54, 29.51, 22.8, 14.3; ^{19}F NMR (376 MHz, $CDCl_3$) δ = −137.7; HRMS−APCI$^+$ (m/z) calcd for $C_{38}H_{53}F_2O_2$ ([M + H]$^+$) 579.4008, found 579.4026.

3,8-Didecyldibenzo[*d,d*']benzo[2,1-*b*:3,4-*b*']difuran (*syn*-DBBDF 5): A 30 mL Schlenk tube was charged with compound **9** (0.57 g, 0.99 mmol), K_2CO_3 (0.30 g, 2.2 mmol), and *N*-methylpyrrolidone (NMP) (10 mL) under argon. The reaction mixture was stirred at 165 °C for 18 h, diluted with EtOAc,

and washed with water. The organic layer was dried over Na_2SO_4, filtered, and concentrated under vacuum. The resulting crude residue was purified by silica gel column chromatography by using hexane/$CHCl_3$ (5:1) (R_f = 0.7) as an eluent to afford 0.49 g (92% yield) of the title compound as a colorless solid: mp 123−127 °C; ^{1}H NMR (400 MHz, $CDCl_3$) δ = 7.89 (d, J = 7.8 Hz, 2H), 7.84 (s, 2H), 7.49 (s, 2H), 7.22 (d, J = 7.8 Hz, 2H), 2.80 (t, J = 7.3 Hz, 4H), 1.75−1.67 (m, 4H), 1.35−1.27 (m, 28H), 0.88 (t, J = 6.9 Hz, 6H); ^{13}C NMR (101 MHz, $CDCl_3$) δ = 157.1, 142.9, 140.8, 124.4, 124.0, 122.4, 120.1, 114.8, 111.7, 36.5, 32.1, 31.9, 29.8, 29.7, 29.51, 29.47, 22.9, 14.3; HRMS−APCI$^+$ (m/z) calcd for $C_{38}H_{51}O_2$ ($[M + H]^+$) 539.3884, found 539.3881. Anal. Calcd for $C_{38}H_{50}O_2$: C, 84.71; H, 9.35. Found: C, 84.05; H, 9.34.

2-(3-Methoxy-6-decylnaphthalen-2-yl)-4,4,5,5-tetramethyl-1,3,2-dioxaborolane (10): A 50 mL Schlenk tube was charged with 2-decyl-7-methoxynaphthalene (0.24 g, 0.79 mmol), *N*,*N*,*N′*,*N′*-tetramethylethylenediamine (0.35 mL, 2.4 mmol), and Et_2O (20 mL) under argon. The mixture was cooled to −78 °C with a dry ice/acetone bath. *s*-BuLi (1.40 mL of 1.06 M hexane solution, 1.5 mmol) was added dropwise at −78 °C. After the reaction mixture was stirred at the same temperature for 2 h, 2-isopropoxy-4,4,5,5-tetramethyl-1,3,2-dioxaborolane (0.52 mL, 2.6 mmol) was added dropwise. The resulting mixture was gradually warmed to room temperature and stirred for 16 h. Water was added to the reaction mixture, and the organic layer was separated. The aqueous layer was extracted with Et_2O, and the combined organic layers were dried over Na_2SO_4, filtered, and concentrated under reduced pressure. The resulting crude residue was purified by silica gel column chromatography by using hexane/AcOEt (5:1) and 1% Et_3N as an eluent to afford 0.15 g (45% yield) of the title compound as a colorless oil: ^{1}H NMR (400 MHz, $CDCl_3$) δ = 8.17 (s, 1H), 7.69 (d, J = 8.2 Hz, 1H), 7.47 (s, 1H), 7.15 (d, J = 8.2 Hz, 1H), 7.01 (s, 1H), 3.92 (s, 3H), 2.72 (t, J = 7.3 Hz, 2H), 1.72−1.64 (m, 2H), 1.38 (s, 12H), 1.33−1.25 (m, 14H), 0.87 (t, J = 6.9 Hz, 3H); ^{13}C NMR (101 MHz, $CDCl_3$) δ = 161.2, 142.3, 138.2, 136.6, 128.2, 127.0, 125.2, 125.1, 119.2, 104.4, 83.7, 55.8, 36.4, 32.0,

31.4, 29.74, 29.72, 29.67, 29.52, 29.46, 24.9, 22.8, 14.3; HRMS−APCI$^+$ (*m*/*z*) calcd for $C_{27}H_{42}BO_3$ ([M + H]$^+$) 425.3222, found 425.3222.

3,3'-(2,3-Difluoro-1,4-phenylene)bis(7-decyl-2-methoxynaphthalene) (11): A 50 mL Schlenk tube was charged with **10** (0.67 g, 1.6 mmol), 2,3-difluoro-1,4-diiodobenzene (0.29 g, 0.78 mmol), $Pd(PPh_3)_4$ (45 mg, 39 μmol), K_3PO_4 (0.82 g, 3.9 mmol), and DMF (15 mL) under argon. The reaction mixture was degassed by three freeze−thaw pump cycles, and the tube was filled with argon. The reaction mixture was stirred at 100 °C for 66 h, diluted with $CHCl_3$, and washed with water. The organic layer was dried over Na_2SO_4, filtered, and concentrated under reduced pressure. The resulting crude residue was purified by silica gel column chromatography by using hexane/EtOAc (5/1) (R_f = 0.7) as an eluent to afford 0.44 g (80% yield) of the title compound as a pale yellow solid; mp 97−100 °C; ^{1}H NMR (400 MHz, $CDCl_3$) δ = 7.76 (s, 2H), 7.71 (d, *J* = 8.2 Hz, 2H), 7.57 (s, 2H), 7.23−7.22 (m, 4H), 7.20 (s, 2H), 3.94 (s, 6H), 2.76 (t, *J* = 7.8 Hz, 2H), 1.72−1.67 (m, 4H), 1.35−1.27 (m, 28H), 0.88 (t, *J* = 6.9 Hz, 6H); ^{13}C NMR (101 MHz, $CDCl_3$) δ = 155.5, 148.7 (dd, *J* = 251.1, 15.3 Hz), 141.8, 134.9, 130.7, 127.8, 127.5 (dd, *J* = 8.6, 4.8 Hz), 127.1, 125.8, 125.2, 125.1, 105.4, 55.8, 36.4, 32.1, 31.6, 29.80, 29.78, 29.73, 29.5, 29.4, 22.9, 14.3; ^{19}F NMR (376 MHz, $CDCl_3$) δ = −138.8; HRMS−APCI$^+$ (*m*/*z*) calcd for $C_{48}H_{61}F_2O_2$ ([M + H]$^+$) 707.4634, found 707.4641.

3,3'-(2,3-Difluoro-1,4-phenylene)bis(7-decylnaphthalen-2-ol) (12): A 50 mL Schlenk tube was charged with compound **11** (0.44 g, 0.62 mmol) and CH_2Cl_2 (15 mL) under argon. To the solution was added BBr_3 (3.7 mL of 1 M CH_2Cl_2 solution) at 0 °C dropwise, and the reaction mixture was stirred at 25 °C for 18 h. After the addition of ice water and saturated aqueous Na_2CO_3, the resulting mixture was extracted with CH_2Cl_2. The combined organic layers were washed with brine, dried over Na_2SO_4, filtered, and concentrated under reduced pressure. The resulting crude residue was purified by silica gel column chromatography by using hexane/EtOAc (3:1) (R_f = 0.4) as an eluent to afford 0.35 g (85%

yield) of the title compound as a colorless solid: mp 191−196 °C; ^{1}H NMR (400 MHz, $CDCl_3$) δ = 7.75 (s, 2H), 7.70 (d, *J* = 8.7 Hz, 1H), 7.48 (s, 1H), 7.30 (s, 2H), 7.23−7.20 (m, 4H), 5.06 (s, 2H), 2.75 (t, *J* = 8.2 Hz, 4H), 1.72−1.69 (m, 4H), 1.35−1.27 (m, 28H), 0.88 (t, *J* = 6.9 Hz, 6H); ^{13}C NMR (101 MHz, $CDCl_3$) δ = 151.0, 149.1 (dd, *J* = 252.1, 15.3Hz), 142.2, 135.2, 130.9, 128.0, 127.6, 127.1 (dd, *J* = 8.6, 5.8 Hz), 126.4, 126.1, 124.8, 122.8, 110.5, 36.4, 32.1, 31.4, 29.8, 29.7, 29.53, 29.49, 22.8, 14.2; ^{19}F NMR (376 MHz, $CDCl_3$) δ = −137.6; HRMS-APCI$^+$ (*m*/*z*) calcd for $C_{46}H_{57}F_2O_2$ ($[M + H]^+$) 679.4321, found 679.4329.

4,11-Didecyldinaphtho[2,3-*d*:2',3'-*d*']benzo[2,1-*b*:3,4-*b*']difurane (*syn*-DNBDF 6): A 30 mL Schlenk tube was charged with compound **12** (0.35 g, 0.52 mmol), K_2CO_3 (0.16 g, 1.2 mmol) and *N*-methylpyrrolidone (NMP, 12 mL) under argon. The reaction mixture was stirred at 165 °C for 18 h. After cooling to room temperature, the crude mixture was precipitated from excess water and filtered to afford 0.29 g (87% yield) of the title compound as a colorless solid: mp > 250 °C; ^{1}H NMR (300 MHz, $CDCl_3$) δ = 8.42 (s, 2H), 8.02 (s, 2H), 7.98−7.95 (m, 4H), 7.76 (s, 2H), 7.35 (dd, *J* = 8.4, 1.7 Hz, 2H), 2.83 (t, *J* = 7.6 Hz, 4H), 1.81-1.71 (m, 4H), 1.38−1.28 (m, 28H), 0.88 (t, *J* = 6.9 Hz, 3H); ^{13}C NMR (126 MHz,$Cl_2CDCDCl_2$, 85 °C) δ = 155.4, 141.6, 140.9, 133.3, 129.0, 128.0, 126.4, 126.0, 125.1, 124.6, 118.7, 115.4, 106.8, 36.0, 31.7, 30.9, 29.4, 29.31, 29.26, 29.1, 22.4, 13.8; HRMS−MALDI$^+$ (*m*/*z*) calcd for $C_{46}H_{54}O_2$ (M^+) 638.4118, found 638.4118. Anal. Calcd for $C_{46}H_{54}O_2$: C, 86.47; H, 8.52. Found: C, 85.92; H, 8.37.

Device fabrication and evaluation

OFET devices with vacuum-deposited thin film were fabricated as follows. Heavily doped n-type Si wafers with a 300 nm-thick layer of thermally grown SiO_2 (C_i = 11.5 nF·cm^{-2}) were used both as the substrate and as the gate electrode. HMDS treatments were carried out by exposing the Si/SiO_2 substrates to HMDS vapor at room temperature in a closed desiccator overnight. The clean Si/SiO_2 substrates or HMDS-treated Si/SiO_2 substrates were placed in an evaporator for organic and metal deposition. Organic thin films (~50 nm thickness) were vacuum-deposited through a shadow mask (500 μm square for each area, channel width W = 500 μm) onto the substrates maintained at various temperatures (T_{sub}) at a deposition rate of 0.4~1 Å·s^{-1} by monitoring with a quartz crystal thickness monitor. The gold thin film (~50 nm thickness) as source and drain electrodes were deposited at a rate of 0.8 Å·s^{-1} through a shadow mask (channel length L = 50 μm) on the organic thin film, forming a top-contact geometry. The characteristics of the OFET devices were evaluated at room temperature in air with a semiconductor parameter analyzer. Field-effect mobility (μ_{FET}) was calculated in the saturation regime of the I_d using the following equation,

$$I_d = (WC_i/2L)\mu_{FET} (V_g - V_{th})^2$$

Where C_i is the capacitance of the SiO_2 insulator, and V_d and V_{th} are the gate and threshold voltages, respectively. Current on/off ratio (I_{on}/I_{off}) was determined from the I_d at V_g = 0 V (I_{off}) and V_g = –50 V (I_{on}). The μ_{FET} data reported are typical values from several different devices.

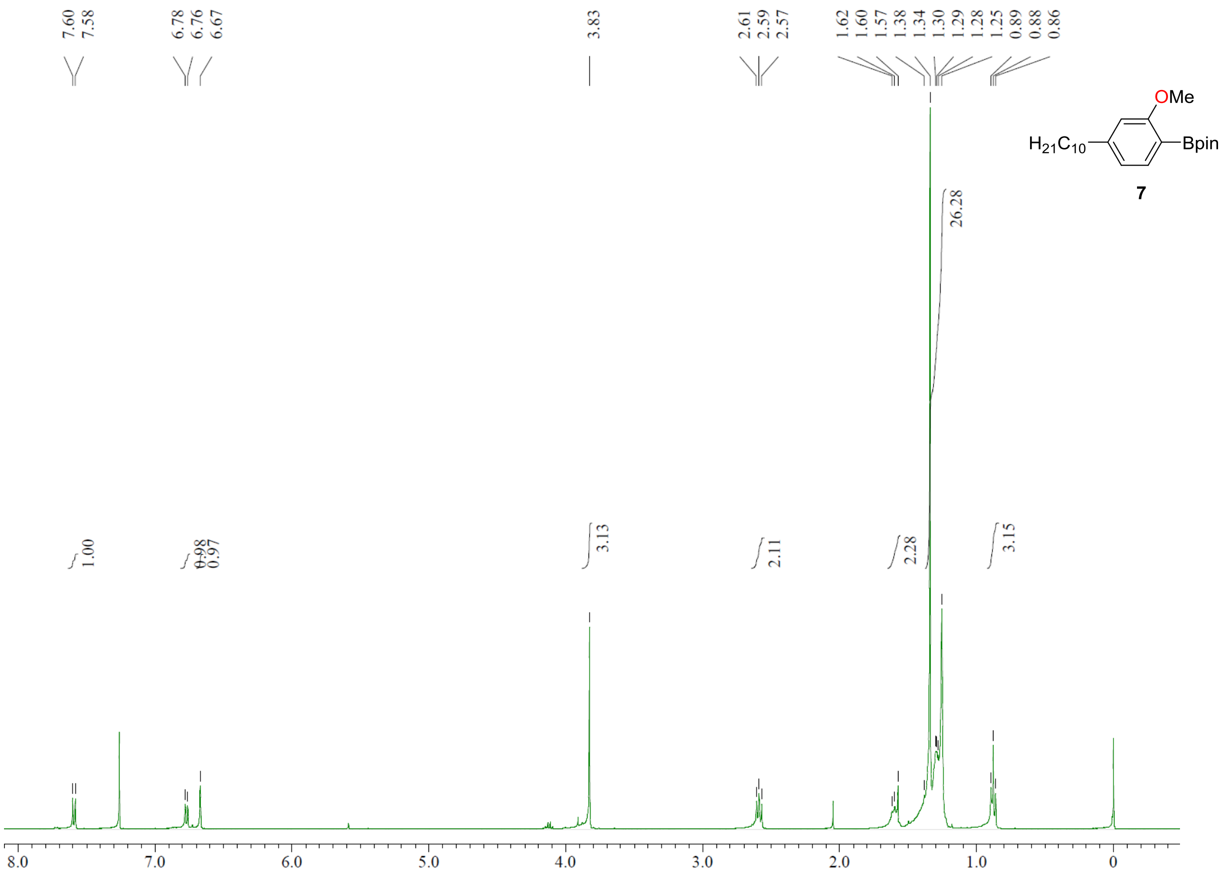

Figure S1. ^{1}H NMR spectrum of **7** ($CDCl_3$).

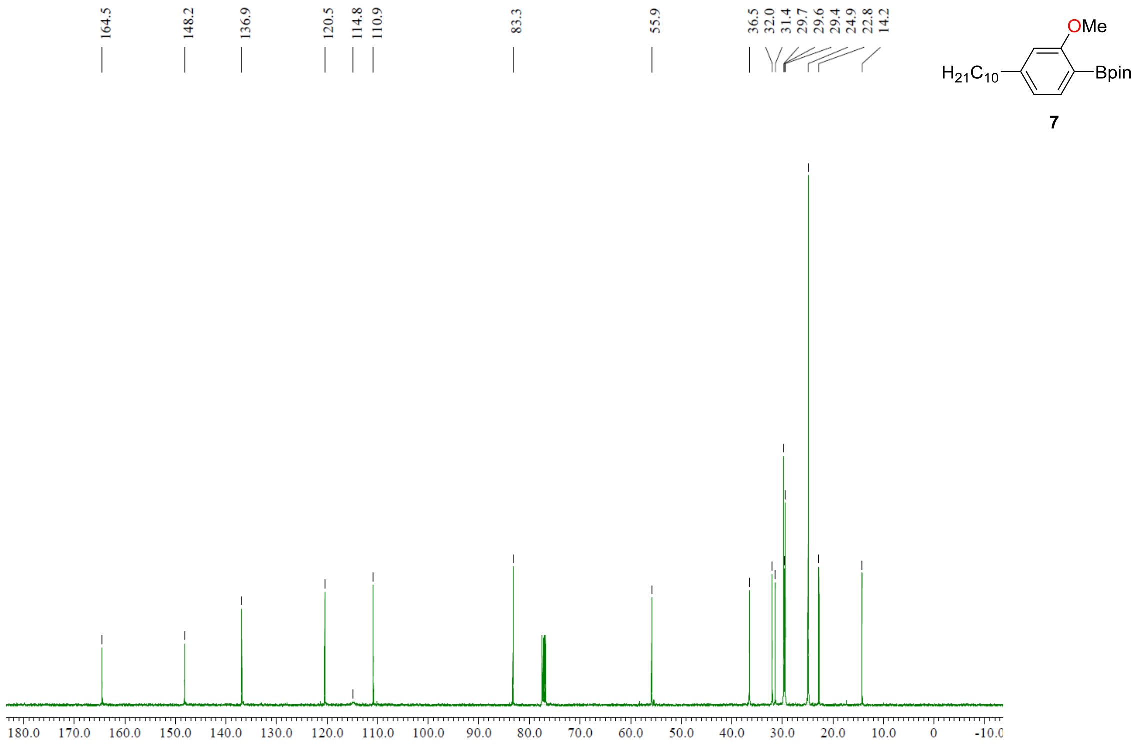

Figure S2. ^{13}C NMR spectrum of **7** ($CDCl_3$).

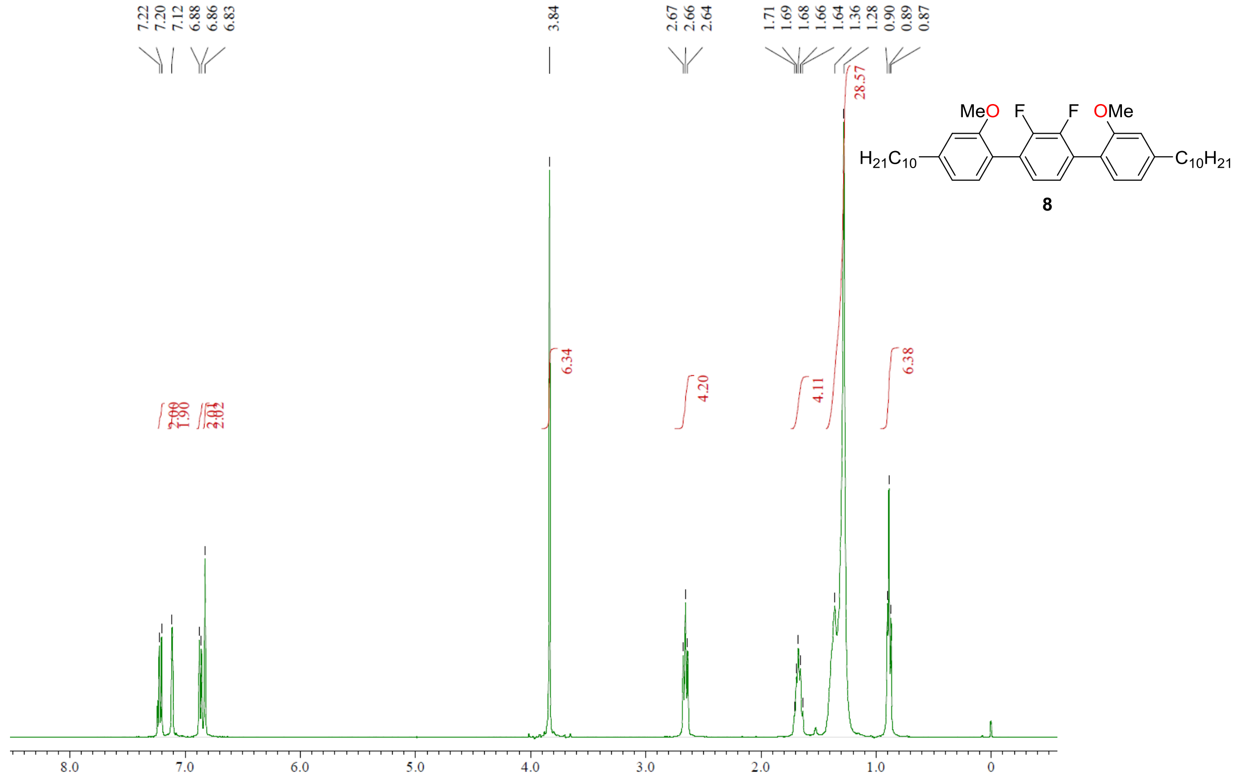

Figure S3. ^{1}H NMR spectrum of **8** ($CDCl_3$).

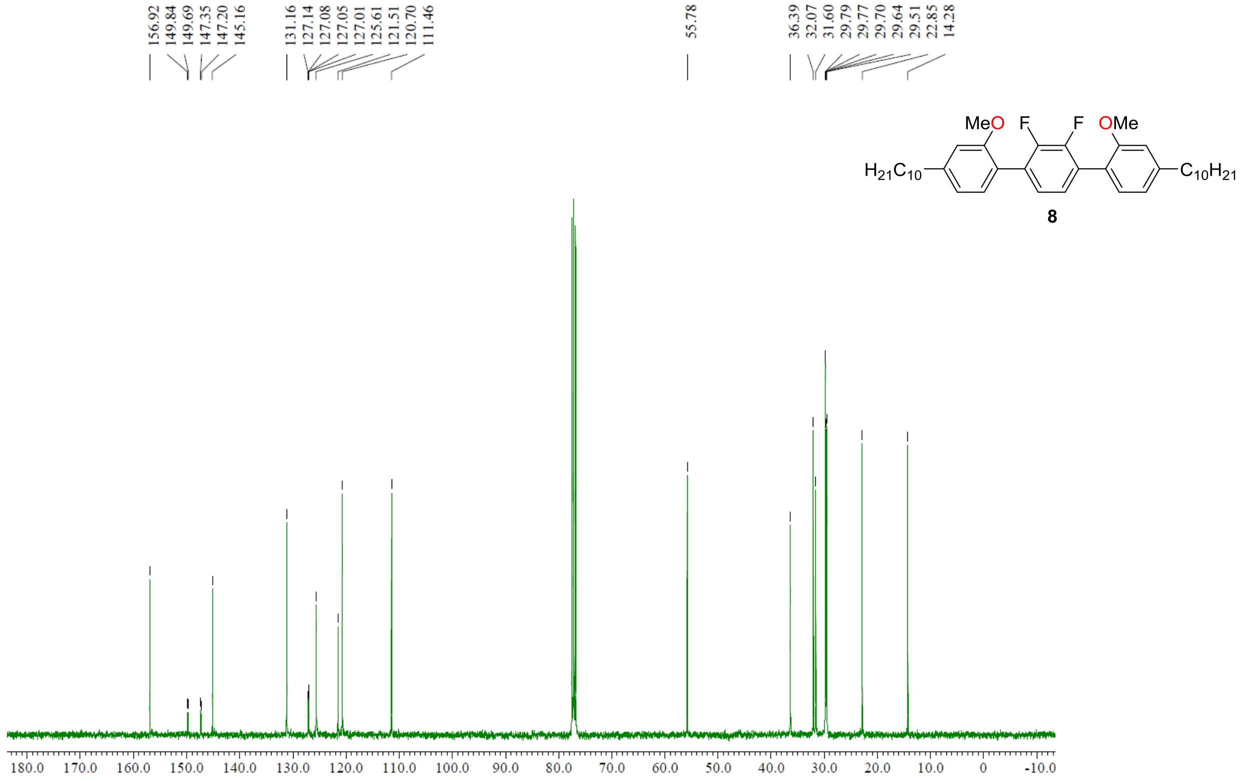

Figure S4. ^{13}C NMR spectrum of **8** ($CDCl_3$).

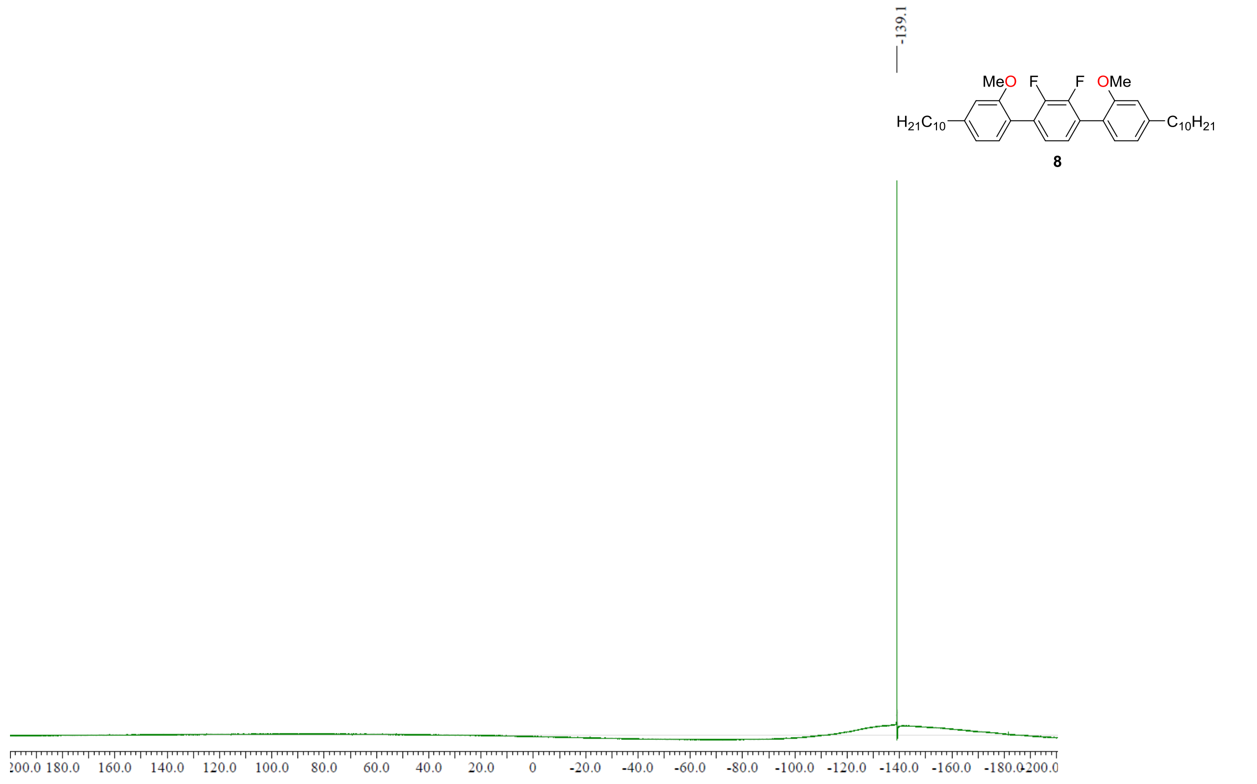

Figure S5. ^{19}F NMR spectrum of **8** ($CDCl_3$).

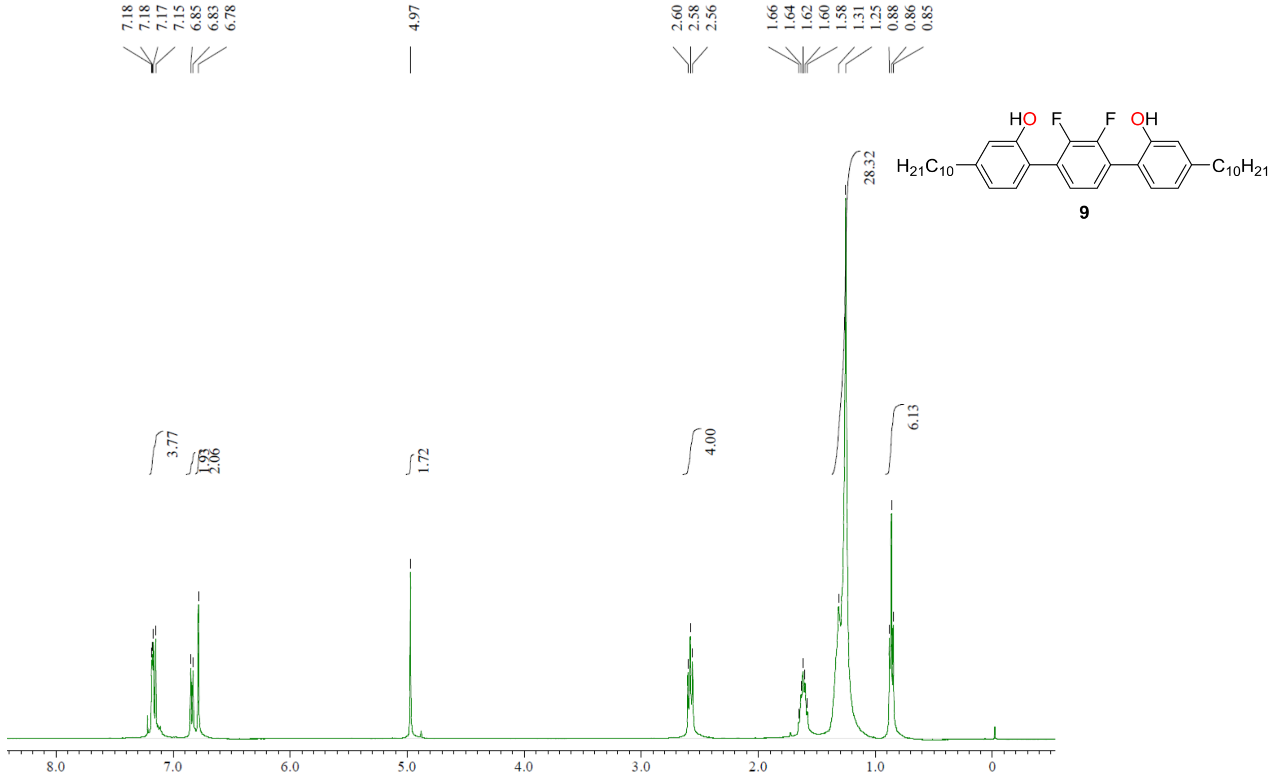

Figure S6. ^{1}H NMR spectrum of **9** ($CDCl_3$).

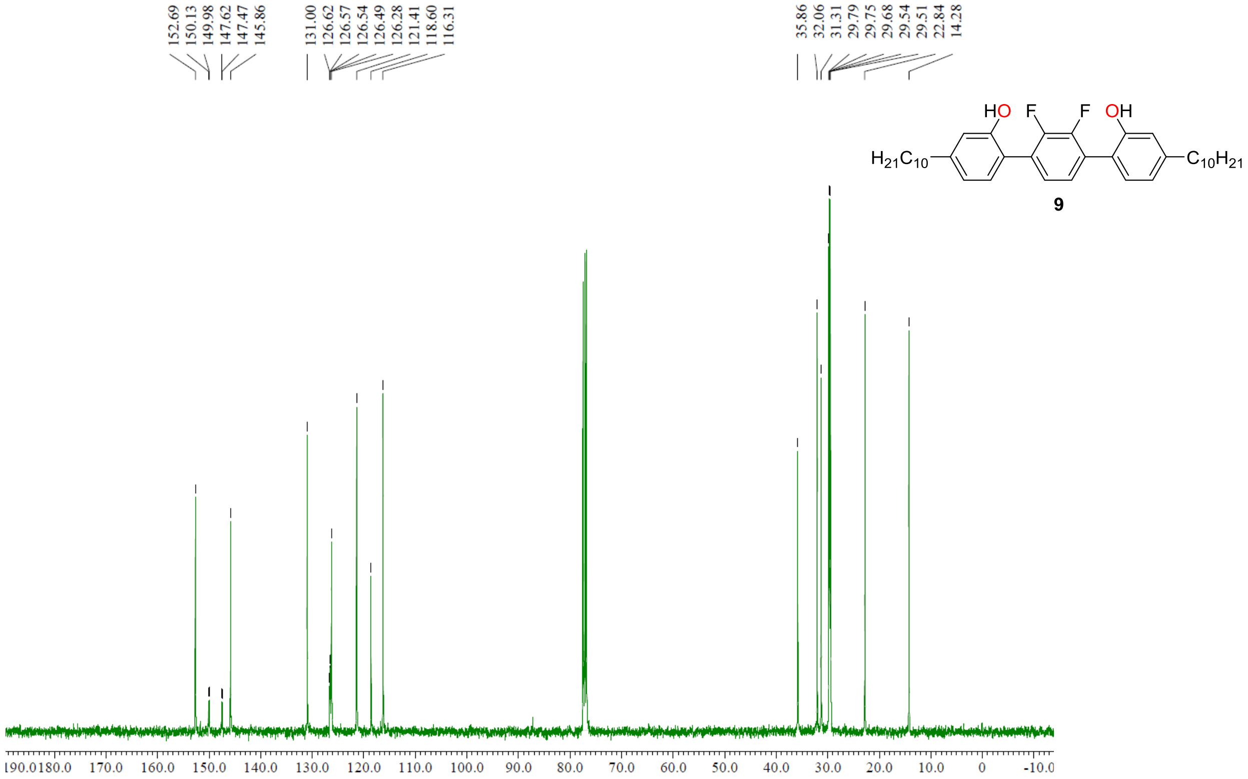

Figure S7. ^{13}C NMR spectrum of **9** ($CDCl_3$).

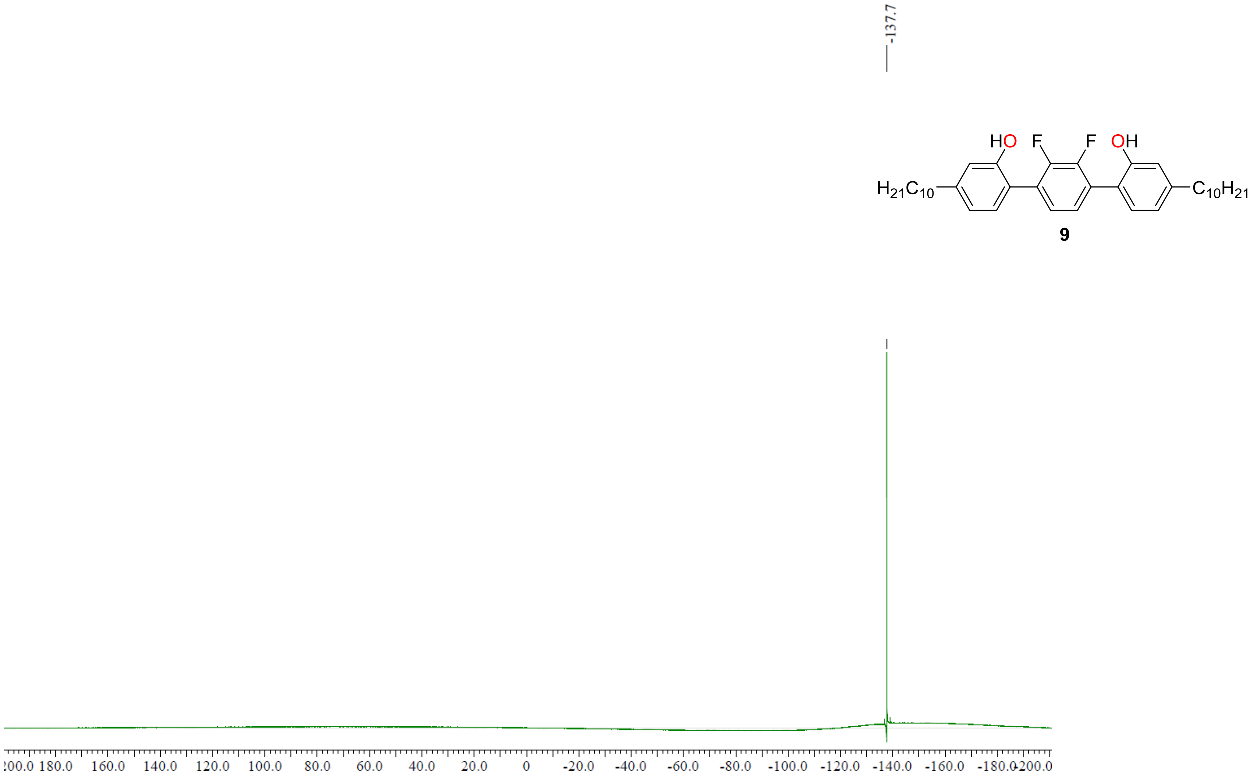

Figure S8. ^{19}F NMR spectrum of **9** ($CDCl_3$).

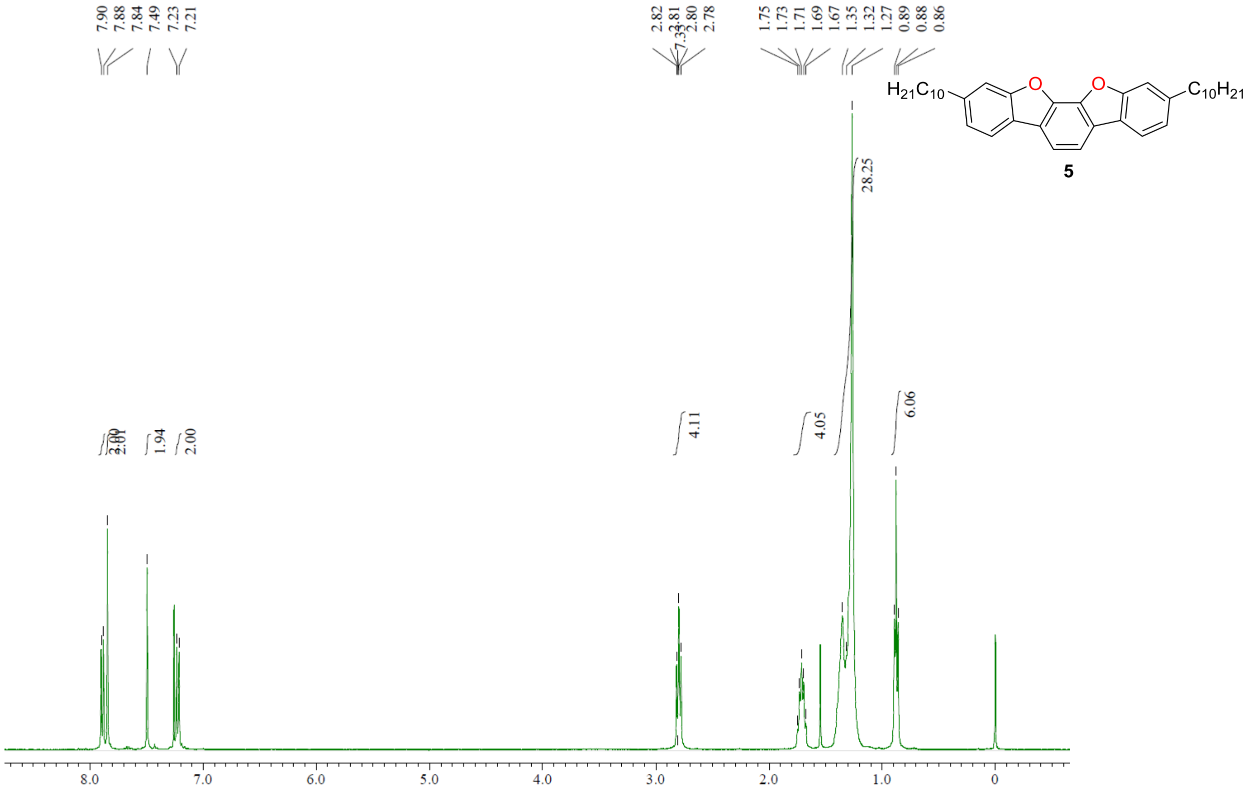

Figure S9. ^{1}H NMR spectrum of **5** ($CDCl_3$).

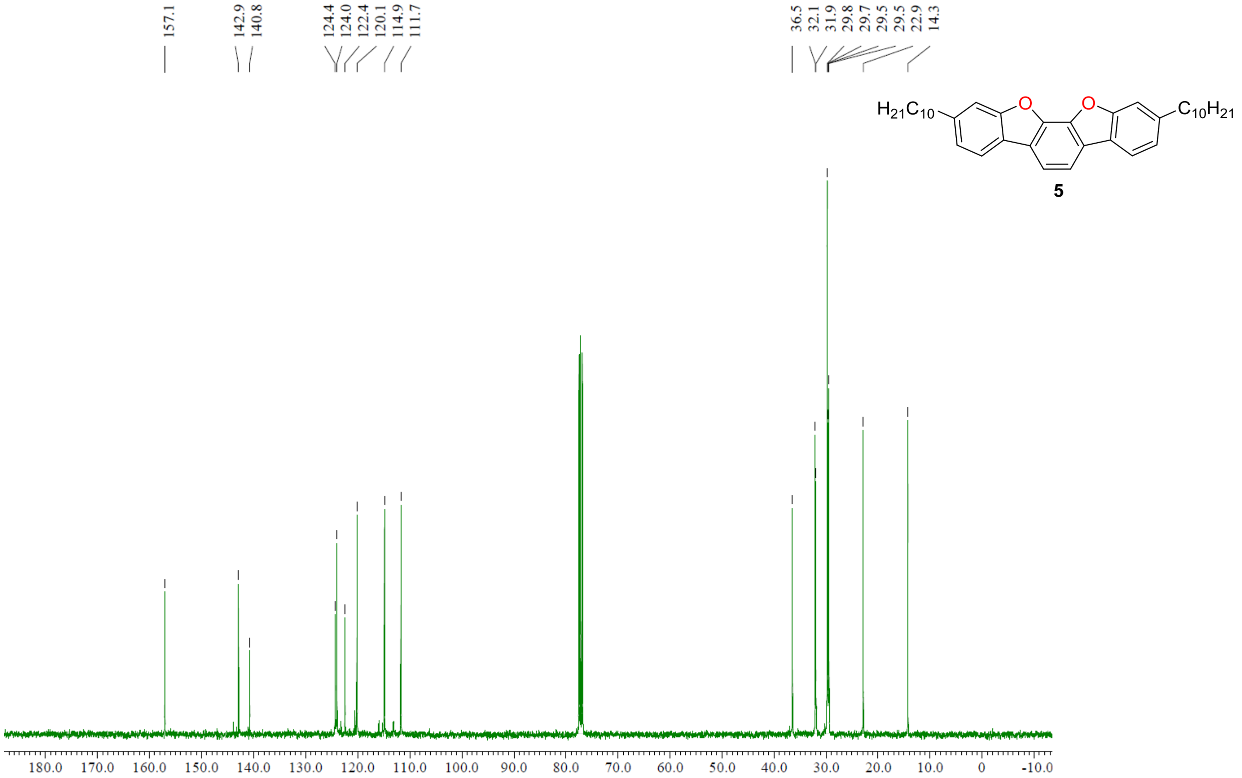

Figure S10. ^{13}C NMR spectrum of **5** ($CDCl_3$).

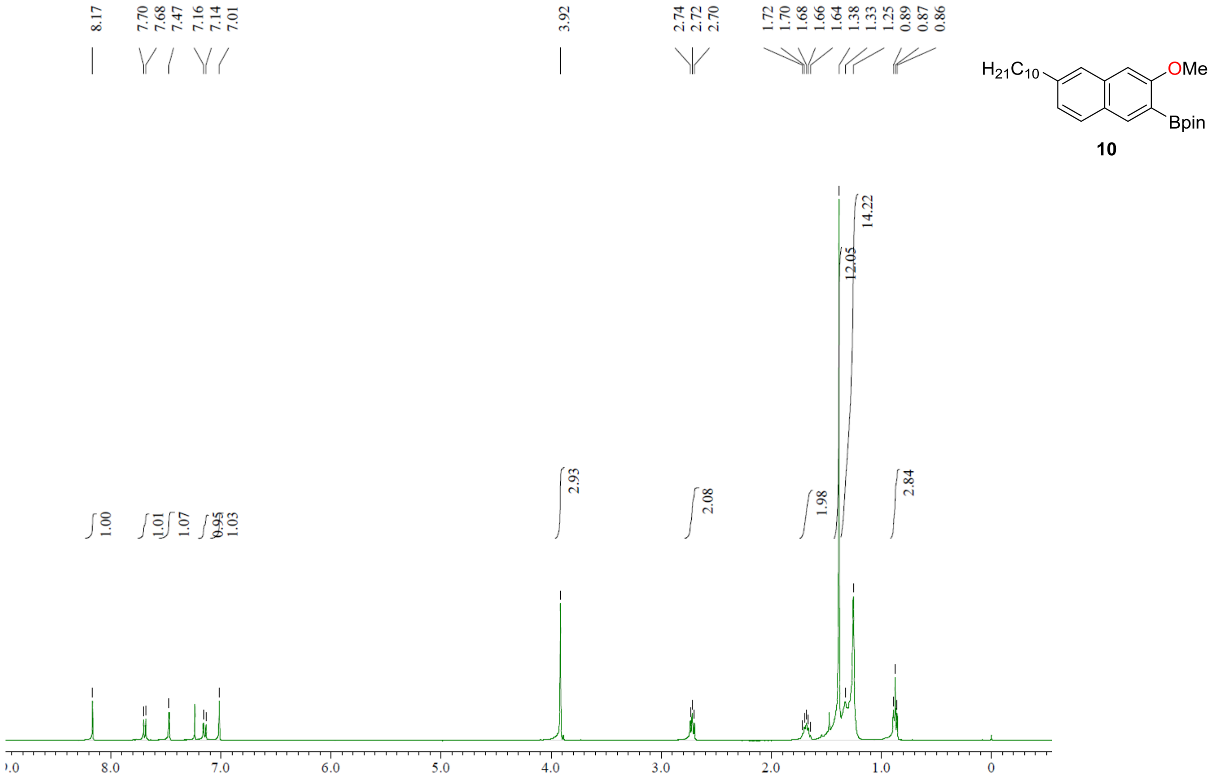

Figure S11. ^{1}H NMR spectrum of **10** ($CDCl_3$).

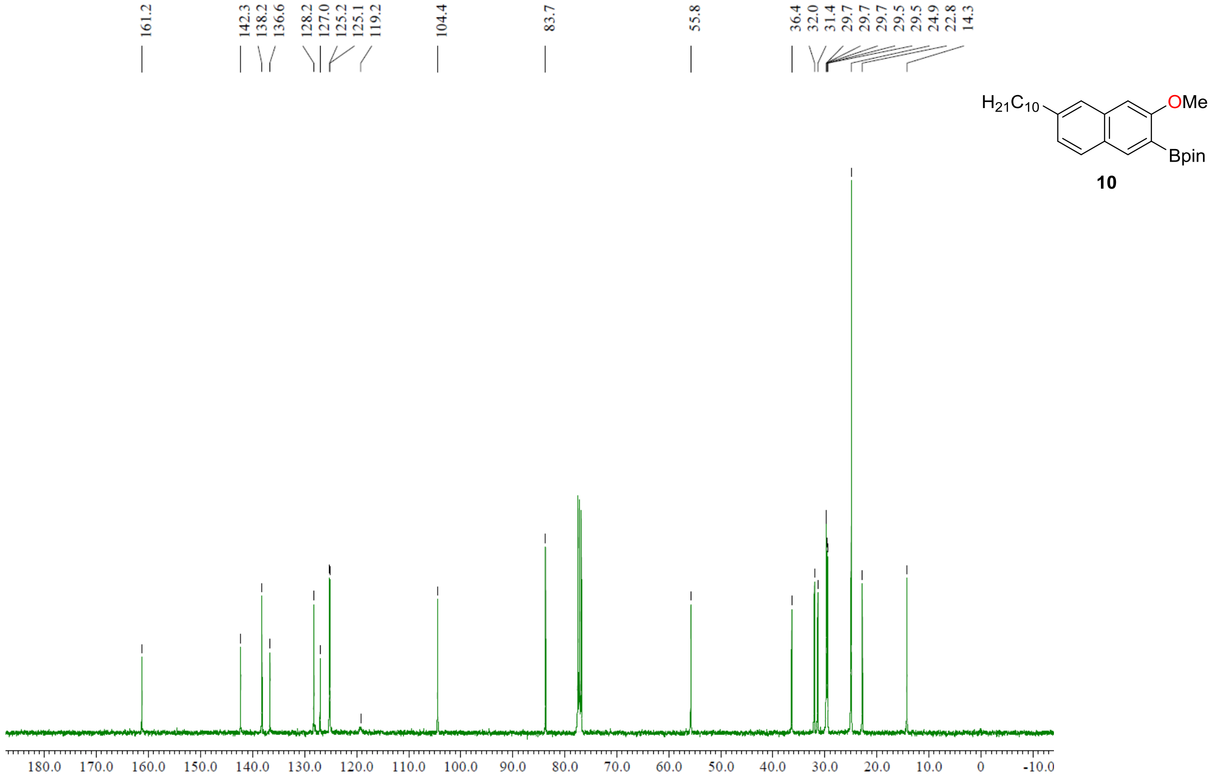

Figure S12. ^{13}C NMR spectrum of **10** ($CDCl_3$).

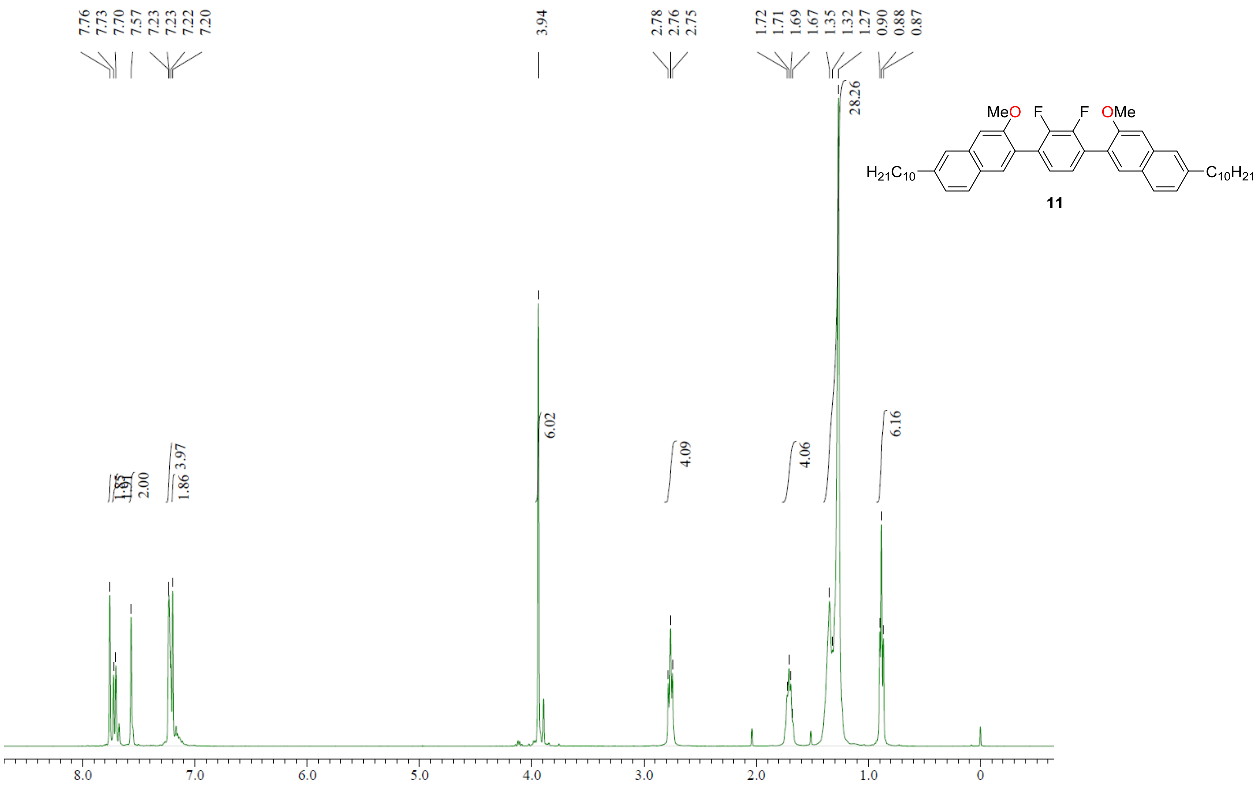

Figure S13. ^{1}H NMR spectrum of **11** ($CDCl_3$).

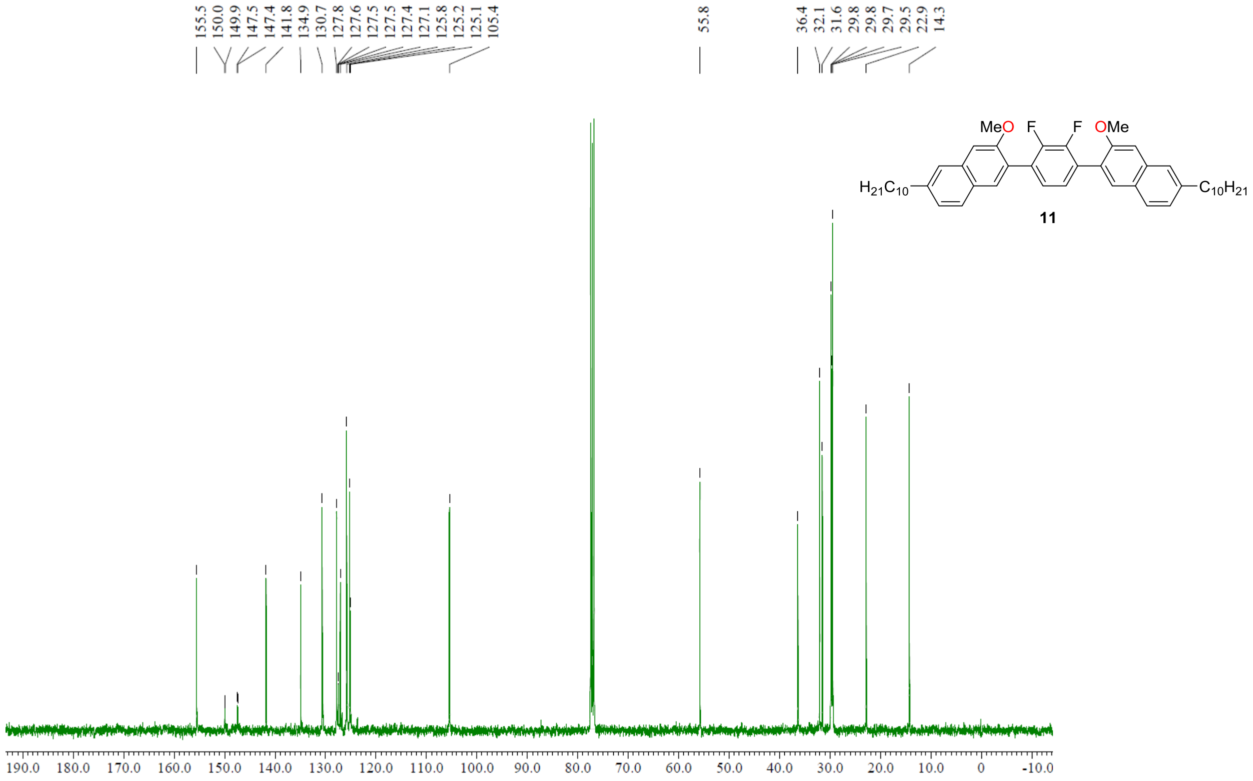

Figure S14. ^{13}C NMR spectrum of **11** ($CDCl_3$).

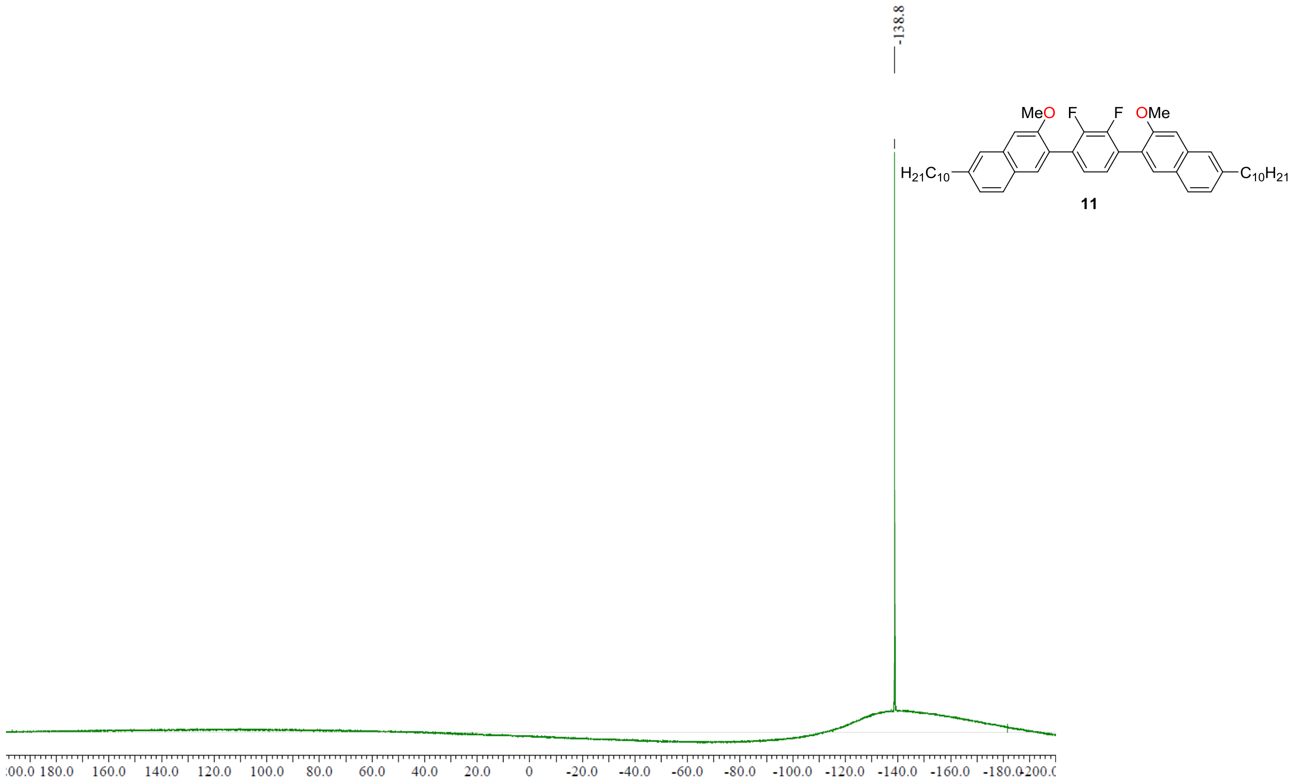

Figure S15. ^{19}F NMR spectrum of **11** ($CDCl_3$).

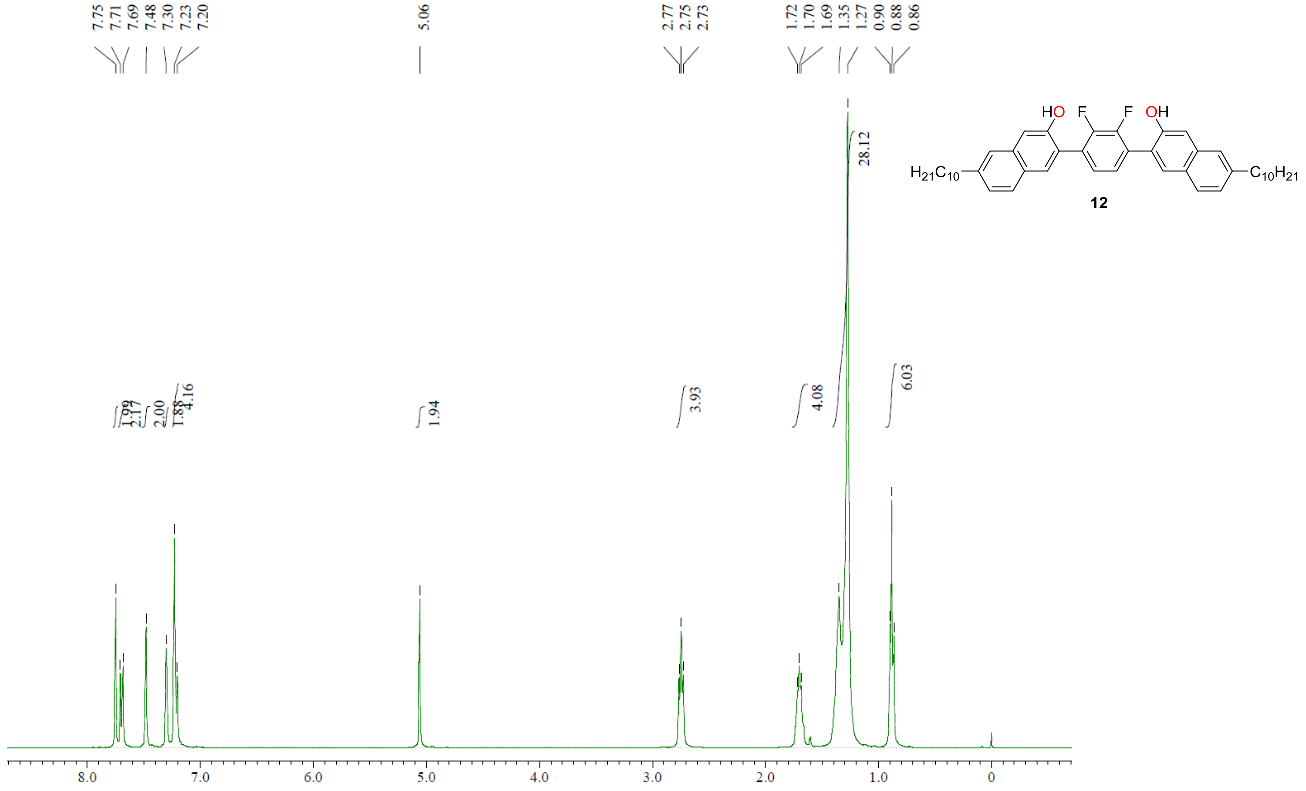

Figure S16. ^{1}H NMR spectrum of **12** ($CDCl_3$).

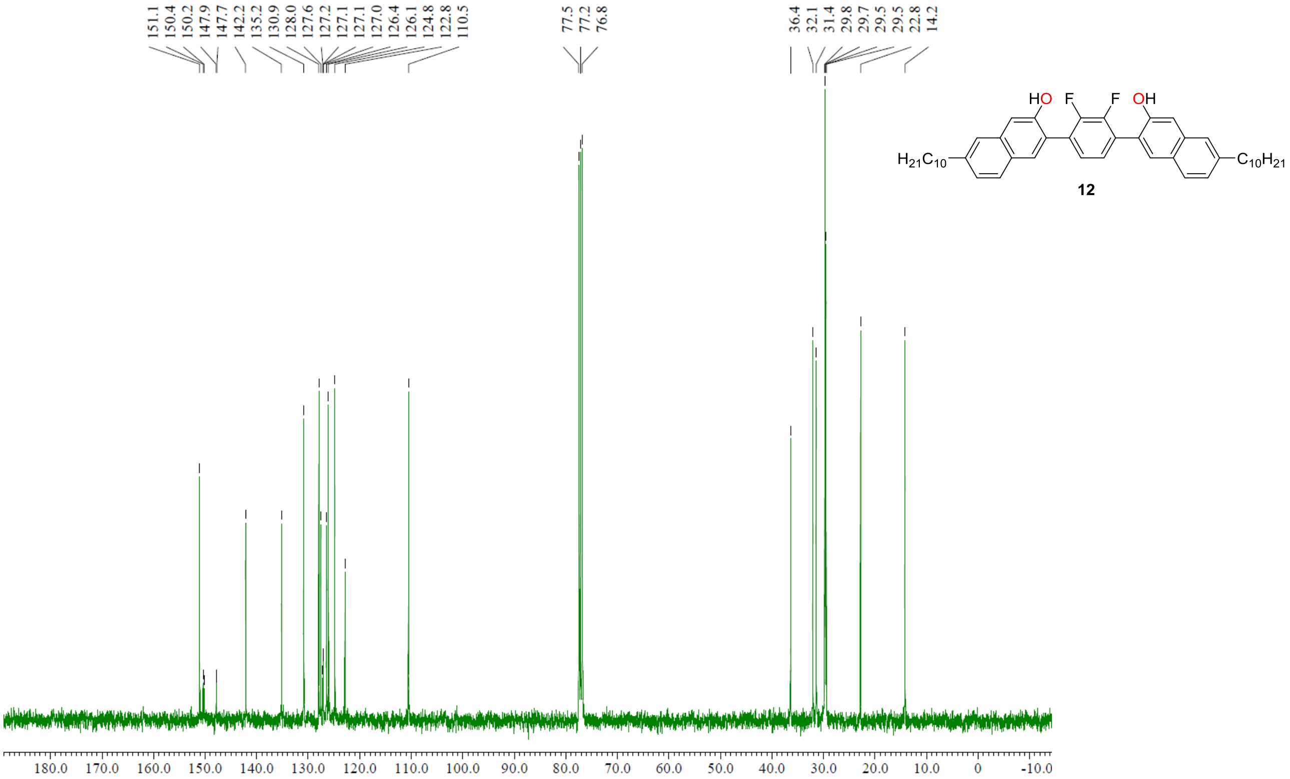

Figure S17. ^{13}C NMR spectrum of **12** ($CDCl_3$).

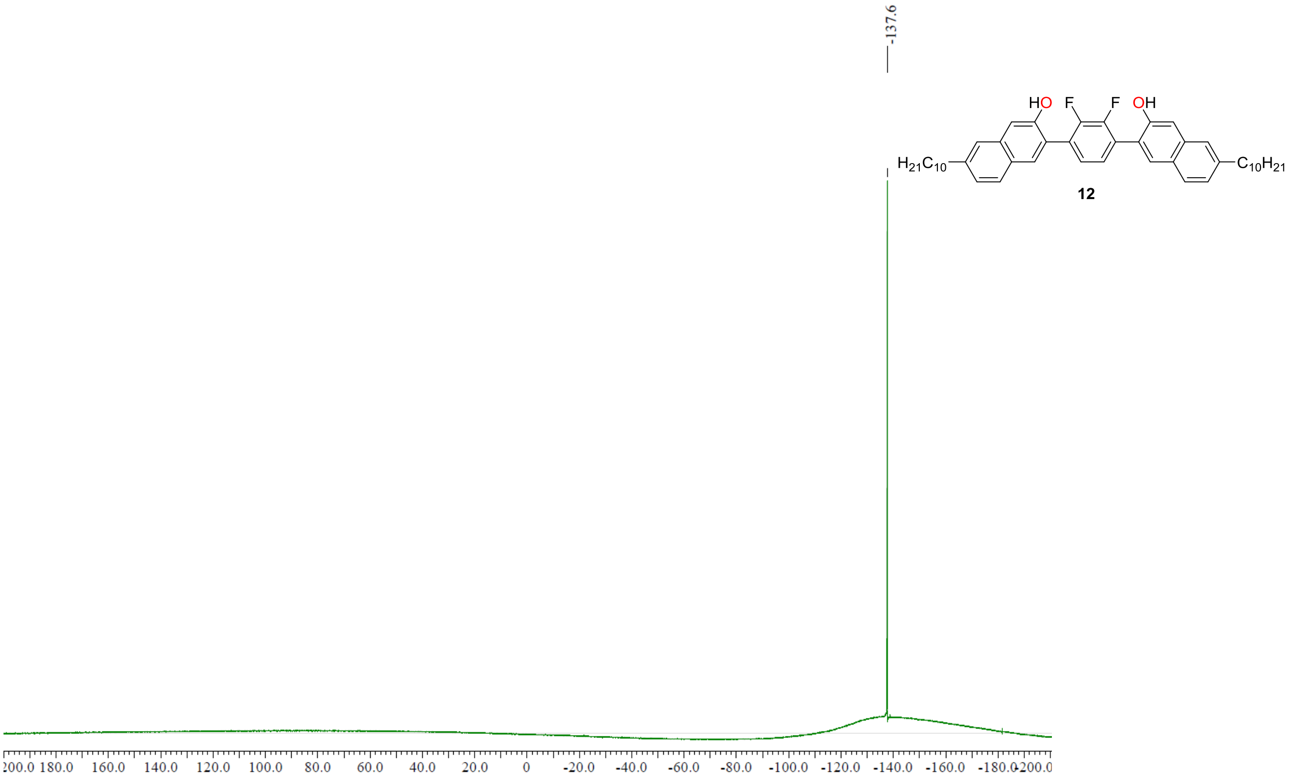

Figure S18. ^{19}F NMR spectrum of **12** ($CDCl_3$).

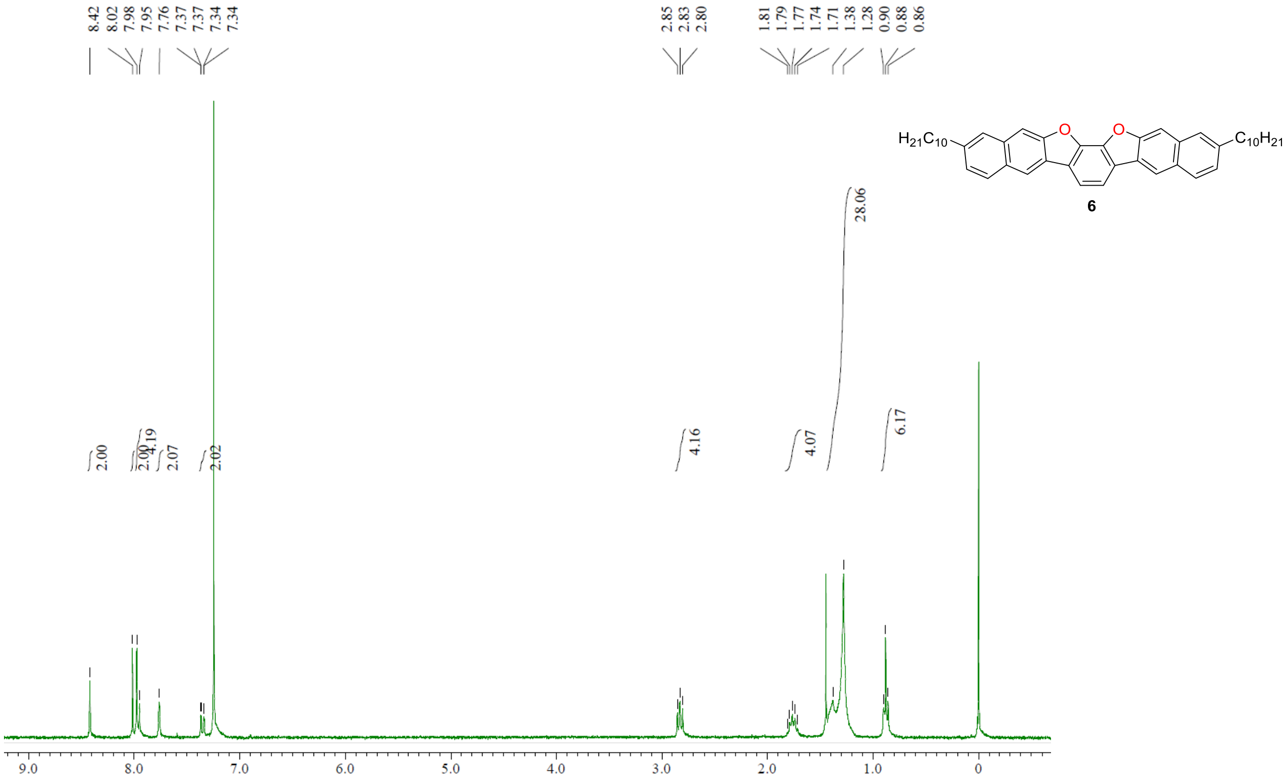

Figure S19. ^{1}H NMR spectrum of **6** ($CDCl_3$).

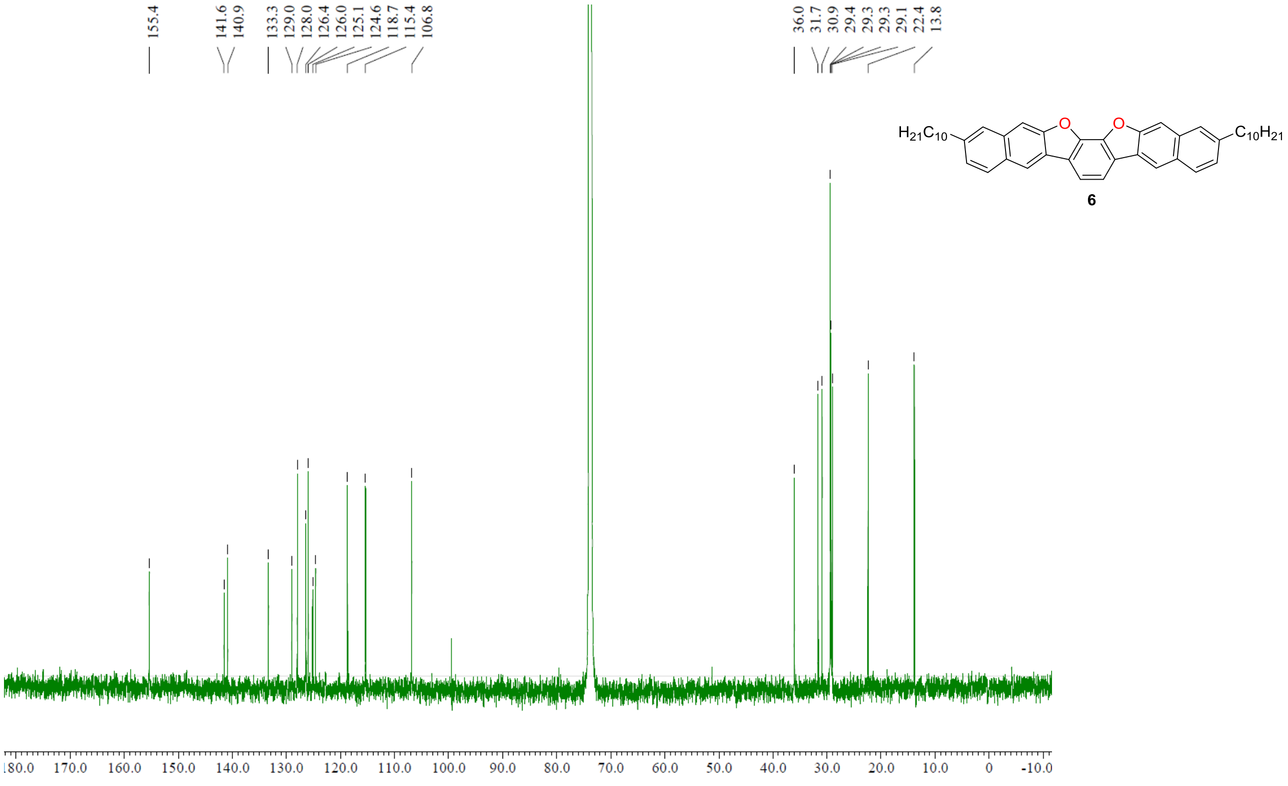

Figure S20. ^{13}C NMR spectrum of **6** ($Cl_2CDCDCl_2$).

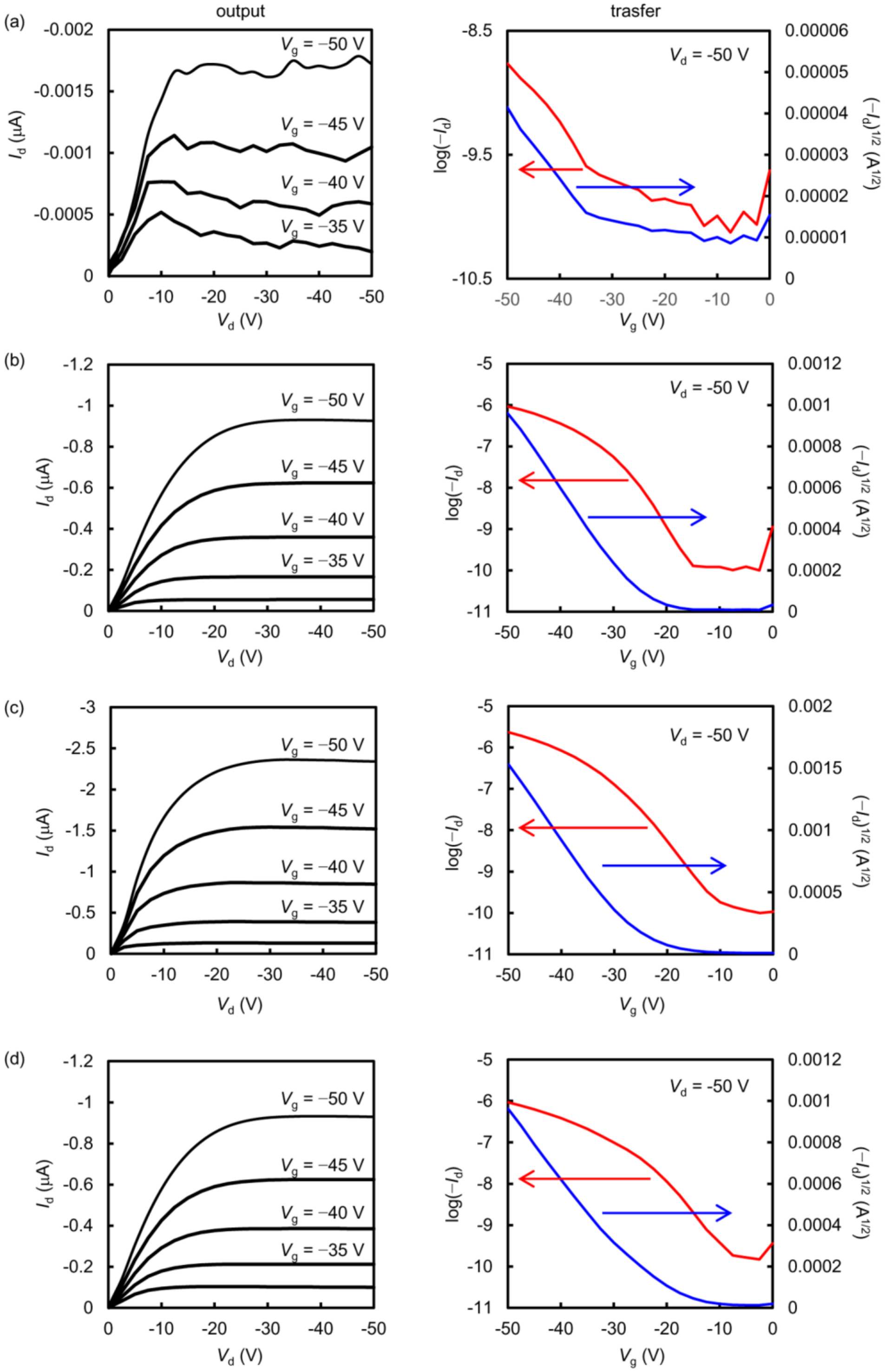

Figure S21. Output and transfer characteristics of the OFETs with a thin film of (a) *syn*-DBBDF **5** (bare Si/SiO_2 substrate; T_{sub} = 30 °C), (b) *syn*-DNBDF **6** (bare Si/SiO_2 substrate; T_{sub} = 30 °C), (c) *syn*-DNBDF **6** (bare Si/SiO_2 substrate; T_{sub} = 90 °C), and (d) *syn*-DNBDF **6** (HMDS-treated Si/SiO_2 substrate; T_{sub} = 30 °C).

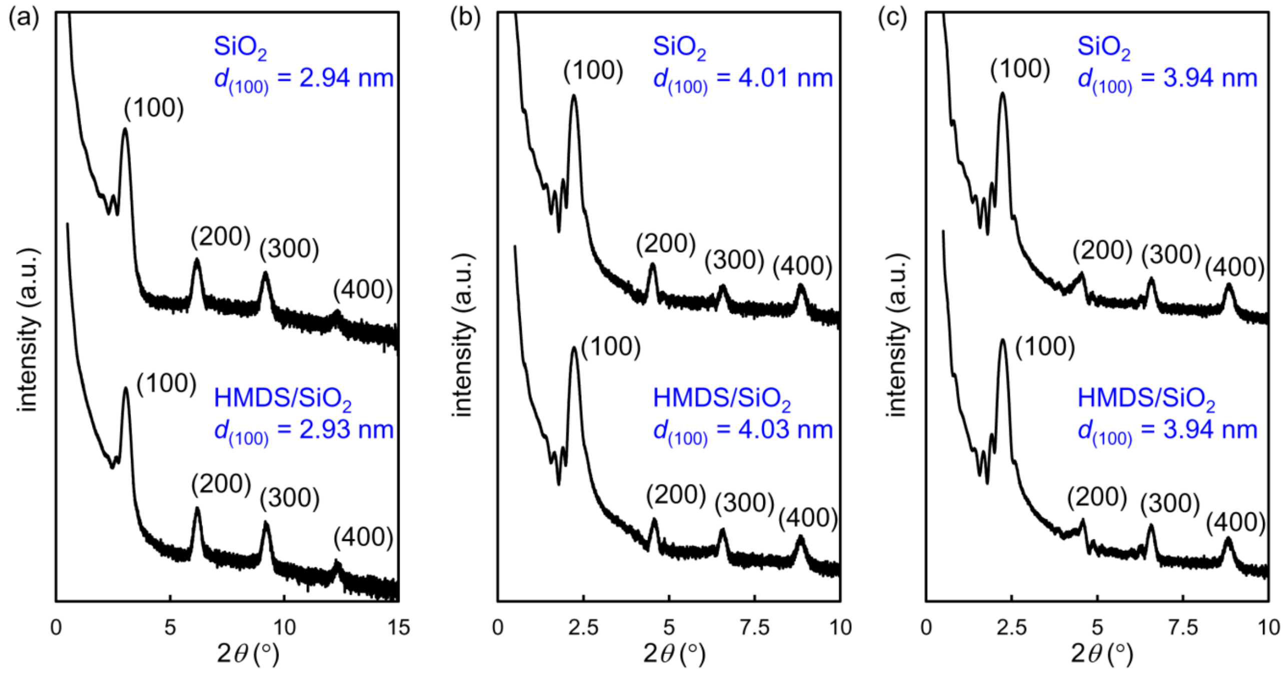

Figure S22. XRD patterns of thin films of (a) *syn*-DBBDF **5** (T_{sub} = 30 °C), (b) *syn*-DNBDF **6** (T_{sub} = 30 °C), and *syn*-DNBDF **6** (T_{sub} = 90 °C).

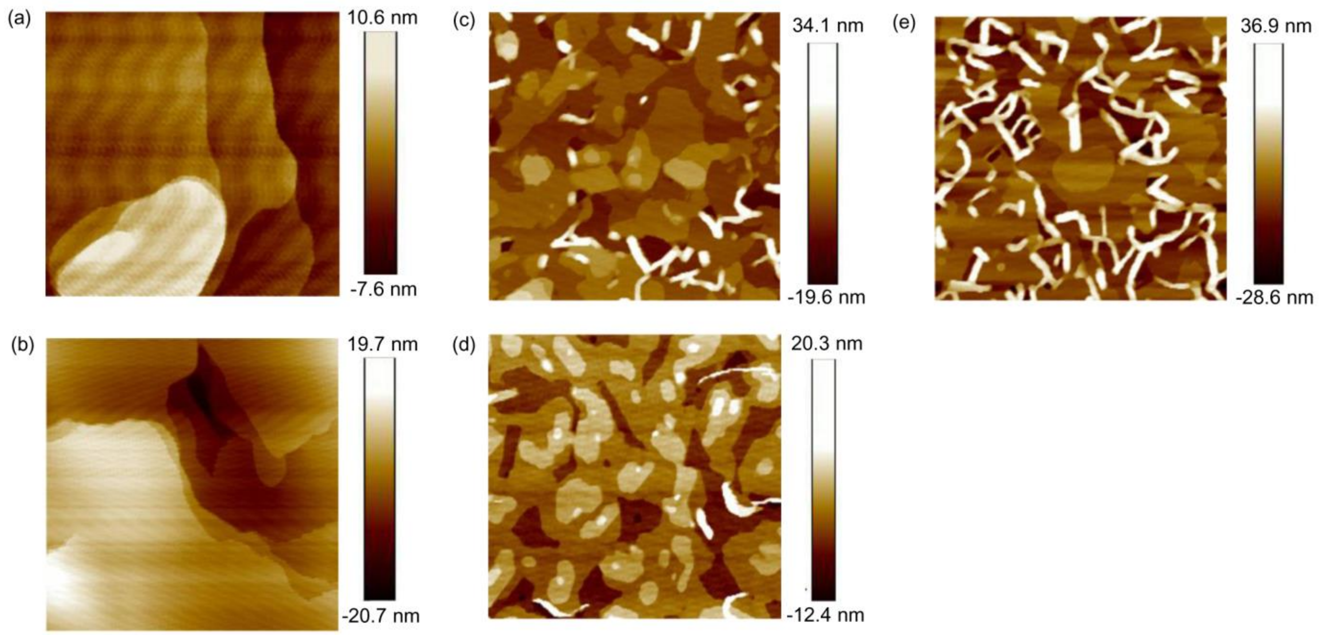

Figure S23. AFM images (2 × 2 μm) of thin films of (a) *syn*-DBBDF **5** (bare Si/SiO_2 substrate; T_{sub} = 30 °C), (b) *syn*-DBBDF **5** (HMDS-treated Si/SiO_2 substrate; T_{sub} = 30 °C), (c) *syn*-DNBDF **6** (bare Si/SiO_2 substrate; T_{sub} = 30 °C), (d) *syn*-DNBDF **6** (bare Si/SiO_2 substrate; T_{sub} = 90 °C), and (e) *syn*-DNBDF **6** (HMDS-treated Si/SiO_2 substrates; T_{sub} = 30 °C).

References

1. Maier, S. K.; Jester, S.-S.; Müller, U.; Müller, W. M.; Höger, S. *Chem. Commun.* **2011**, *47*, 11023.

2. Cardona, C. M.; Li, W.; Kaifer, A. E.; Stockdale, D.; Bazan, G. C. *Adv. Mater.* **2011**, *23*, 2367-2371.

3. Johansson, T.; Mammo, W.; Svensson, M.; Andersson, M. R.; Inganäs, O. *J. Mater. Chem*. **2003**, *13*, 1316.

4. Pommerehne, J.; Vestweber, H.; Guss, W.; Mahrt, R. F.; Bässler, H.; Porsch, M.; Daub, J. *Adv. Mater.* **1995**, *7*, 551-554.
